# The Utility of Human Milk Oligosaccharides against Group B *Streptococcus* Infections of Reproductive Tissues and Cognate Adverse Pregnancy Outcomes

**DOI:** 10.1021/acscentsci.3c00101

**Published:** 2023-08-09

**Authors:** Rebecca E. Moore, Sabrina K. Spicer, Jacky Lu, Schuyler A. Chambers, Kristen N. Noble, Jonathan Lochner, Rebecca C. Christofferson, Karla A. Vasco, Shannon D. Manning, Steven D. Townsend, Jennifer A. Gaddy

**Affiliations:** † Department of Medicine, Vanderbilt University Medical Center, Nashville, Tennessee 37232, United States; ‡ Department of Veterans Affairs, Tennessee Valley Healthcare Systems, Nashville, Tennessee 37212, United States; § Department of Chemistry, Vanderbilt University, Nashville, Tennessee 37240, United States; ∥ Department of Pathology, Microbiology and Immunology, Vanderbilt University Medical Center, Nashville, Tennessee 37232, United States; ⊥ Department of Chemistry, Stanford University, Stanford, California 94305, United States; # Department of Pediatrics, Vanderbilt University Medical Center, Nashville, Tennessee 37232, United States; ○ Department of Pathobiological Sciences, School of Veterinary Medicine, Louisiana State University, Baton Rouge, Louisiana 70803, United States; ◆ Department of Microbiology and Molecular Genetics, Michigan State University, East Lansing, Michigan 48824, United States

## Abstract

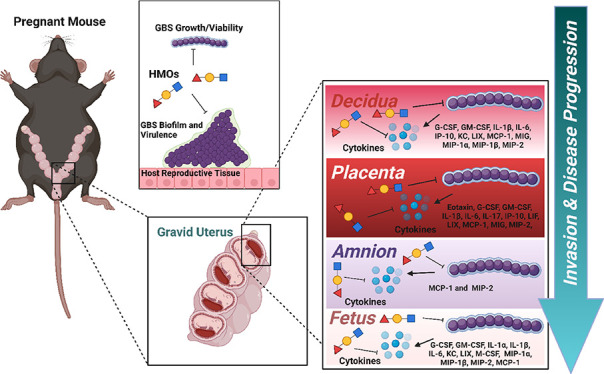

Preterm birth affects nearly 10% of all pregnancies in the United States, with 40% of those due, in part, to infections. *Streptococcus agalactiae* (Group B *Streptococcus*, GBS) is one of the most common perinatal pathogens responsible for these infections. Current therapeutic techniques aimed to ameliorate invasive GBS infections are less than desirable and can result in complications in both the neonate and the mother. To this end, the need for novel therapeutic options is urgent. Human milk oligosaccharides (HMOs), an integral component of human breast milk, have been previously shown to possess antiadhesive and antimicrobial properties. To interrogate these characteristics, we examined HMO-mediated outcomes in both *in vivo* and *ex vivo* models of GBS infection utilizing a murine model of ascending GBS infection, an EpiVaginal human organoid tissue model, and *ex vivo* human gestational membranes. Supplementation of HMOs resulted in diminished adverse pregnancy outcomes, decreased GBS adherence to gestational tissues, decreased colonization within the reproductive tract, and reduced proinflammatory immune responses to GBS infection. Taken together, these results highlight the potential of HMOs as promising therapeutic interventions in perinatal health.

## Abstract 2

Human breast milk sugars reduce bacterial invasion and disease progression of perinatal pathogen Group B *Streptococcus* in an *in vivo* mouse model of disease during pregnancy.

## Introduction

Every year, approximately 11.1% or 15 million babies are born prematurely worldwide, putting both the mother and infant at higher risk of morbidity and mortality due to complications during labor and delivery.^1^ There are often a number of contributing factors resulting in preterm birth (less than 37 weeks gestation); however, chorioamnionitis is frequently associated as the major cause. This inflammation of the fetal membranes is most commonly caused by ascending bacterial infections that develop in the lower genital tract. As the bacteria traverses into the fetal space, the inflammatory response is initiated, often leading to the onset of labor and preterm premature rupture of the membranes (PPROM).^2^

*Streptococcus agalactiae* or Group B *Streptococcus* (GBS) is a frequent commensal bacterium of the human gastrointestinal and reproductive tracts. As an opportunistic pathogen, GBS infections become especially problematic during pregnancy, colonizing up to 30% of pregnant persons. GBS rarely infects healthy individuals but is associated with a number of adverse pregnancy outcomes including preterm birth, stillbirth, neonatal sepsis, pneumonia, and meningitis.^3−6^ Early onset infections are most commonly a result of ascending infection from the vaginal tract ultimately, crossing the placental membranes while *in utero* or from vertical transmission as the baby passes through the birth canal during delivery.^7,8^ Additionally, horizontal transmission can occur nosocomially, through skin-to-skin contact with mother, hospital worker, or community contacts.^8^ Without an effective vaccine in development against GBS infections, the American College of Obstetricians and Gynecologists currently recommends rectovaginal screening between 36- and 37-weeks gestation and encourages the administration of intrapartum antibiotic prophylaxis (IAP) to those who test positive for GBS.^9^ While IAP administration has significantly decreased the incidences of early onset infections, GBS remains a major cause of preterm birth, stillbirth, and miscarriages, as well as late-onset infections.^6,10^ With the emergence of antibiotic resistant strains and the adverse effects associated with antibiotics on the infant’s microflora, new strategies must be employed to prevent and combat these perinatal infections.

The pathogenesis of GBS disease is dependent on the adherent ability of the bacterium to colonize and persist within the host tissues through the formation of biofilms. These structured colonies of microorganisms are found within a self-produced extracellular polymeric matrix (EPS) that adheres to both living and abiotic surfaces. The fully hydrated EPS is comprised mostly of polysaccharides, proteins, lipids, and extracellular DNA and held tightly together through hydrogen bonding.^11,12^ The sialic acid-rich capsular polysaccharides (CPS) not only provide protection from the host immune system, but they have been shown to mediate biofilm formation.^13^ Additionally, the cell-wall-anchored type 2a pili are crucial appendages necessary for GBS adherence and biofilm formation. Together, these components allow the bacterium to thrive in hostile environments such as the nutrient-depleted, acidic vaginal tract of pregnant persons.

In response to the accelerated emergence of multidrug-resistant bacteria, there is an urgent need to develop alternative strategies to minimize this threat. The use of antimicrobial adjuvants, compounds that are administered in combination with an antibiotic, has been an effective strategy. We centered our research on the hypothesis that human milk oligosaccharides (HMOs), a diverse group of carbohydrates present in high quantities in breast milk, possess antimicrobial and antivirulence properties. We discovered that a heterogeneous mixture of HMOs possesses potent bacteriostatic and antibiofilm activity against several pathogens including GBS.^14−16^ And while we observed that no single-entity HMOs were able to inhibit biofilm production in GBS, we discovered that we could convert these HMOs to antibiofilm compounds by incorporating a positive charge on the molecule. Indeed, we found that the four β-amino HMOs we synthesized significantly inhibited biofilm formation in both GBS and methicillin-resistant *Staphylococcus aureus* (MRSA).^17^

In an effort to better understand the innate immune response provoked by GBS infection, we have developed an *ex vivo* model of human extraplacental gestational membrane (EPM) tissue-bacterial infection. We have previously demonstrated that gram-positive pathogens can adhere to and form robust biofilms on the choriodecidual face of the membranes surrounding the developing fetus and induce production of proinflammatory cytokines.^18^ Additionally, we sought to understand host-GBS interactions in our *in vivo* mouse model, which mimics colonization and ascension of GBS throughout the reproductive tract. We hypothesized that with our coculture model HMOs would inhibit GBS adherence and biofilm formation on EpiVaginal human organoid tissue and EPMs collected from healthy, term, nonlaboring C-section placentas, and HMOs would reduce GBS colonization in the mouse model, reducing the incidences of adverse pregnancy outcomes. These results taken together will provide useful information on how we can inhibit GBS ascending infection into the gestational membranes and the prevention of chorioamnionitis.

## Results

### HMOs Prevent GBS Adherence to and Biofilm Formation on EpiVaginal Tissues

**Figure 1:**
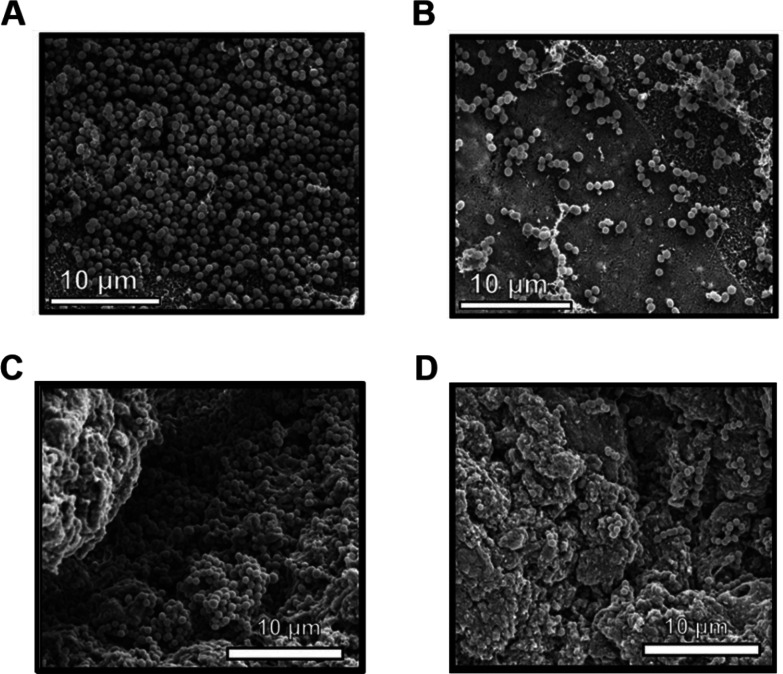
HMOs prevent GBS adherence to and biofilm formation on EpiVaginal tissues and human gestational membranes. High-resolution field-emission gun scanning electron microscopy (FEG-SEM) analyses of GBS strain GB00590 adherence to vaginal tissues and human gestational tissues. FEG-SEM imaging of GBS adherence was performed on GB00590 samples grown with vaginal tissues in medium alone (A), or medium supplemented with 5 mg/mL of HMOs (B); and GB00590 samples grown with human gestational membranes in medium alone (C), or medium supplemented with 5 mg/mL of HMOs (D). The addition of HMOs significantly inhibits GBS adherence. Micrographs were collected at 10,000× magnification, and magnification bars indicate 10 μm. Micrographs are representative of analyses performed in a blinded fashion on tissues derived from 3 biological replicates.

To interrogate GBS interactions with the vaginal epithelium upon supplementation with HMOs, we utilized a reconstructed EpiVaginal human organoid tissue model. We hypothesized that HMOs would limit the biofilm formation and GBS adherence to the luminal tissue surface. GBS (GB590) was cocultured with these tissues for 24 h in either Todd Hewitt Broth (THB) medium alone or with a pooled mixture of HMOs (5 mg/mL). This concentration resides at the low end of physiological relevant concentrations of HMOs naturally present in human breast milk.^19−21^ The tissues were analyzed by field-emission gun scanning electron microscopy (FEG-SEM). In medium alone, GBS forms globular microcolonies, easily adhering to the vaginal epithelium (Figure 1A). Treatment with HMOs significantly reduced GBS adherence and truncated the biofilm architecture (Figure 1B). These results indicate that HMOs are able to prevent vaginal colonization, which is a critical step in the enhancement of GBS disease. To further interrogate the inflammatory response in response to GBS infection in these organoid tissues, multiplex cytokine analyses were performed (Figure S9). Of the 16 cytokines analyzed, we observed an increase in IL-1Rα with respect to GBS infection, while HMO supplementation reversed this increase. We also observed a similar trend (although not significant) in TNF-α. While we did not observe quite the significant changes in proinflammatory cytokine production in the EpiVaginal organoid tissues as we observed in the mouse tissues, this was expected because *ex vivo* tissues tend to have more subtle responses to GBS compared to vertebrate models with full immune responses.

### HMOs Inhibit GBS Adherence and Biofilm Formation to Gestational Membranes

We have previously demonstrated that GBS adheres to and promotes biofilm formation on human extraplacental gestational membranes (EPMs).^22^ To further examine whether HMOs could disrupt GBS adherence and biofilm formation, GBS (GB590) was cocultured with these *ex vivo* human fetal tissues for 24 h in THB medium alone or with a pooled mixed of HMOs (5 mg/mL). The tissues were visualized using FEG-SEM to evaluate changes in biofilm morphology. Just as was observed with the EpiVaginal tissue model, treatment with HMOs significantly reduced bacterial adherence and colonization of GBS to the maternal choriodecidual face of the EPM (Figure 1C–D). We further evaluated bacterial adherence to EPMs in five diverse GBS strains by quantitative culture in response to HMO supplementation, observing a significant decrease in colony forming units (CFUs) in four of the five strains (Figure S3). Taken together, these results point toward limiting the capacity of GBS to penetrate and proliferate within the reproductive tissues.

### HMOs Reduce GBS Pathogenesis and Cognate Disease Outcomes Associated with Infection

**Figure 2:**
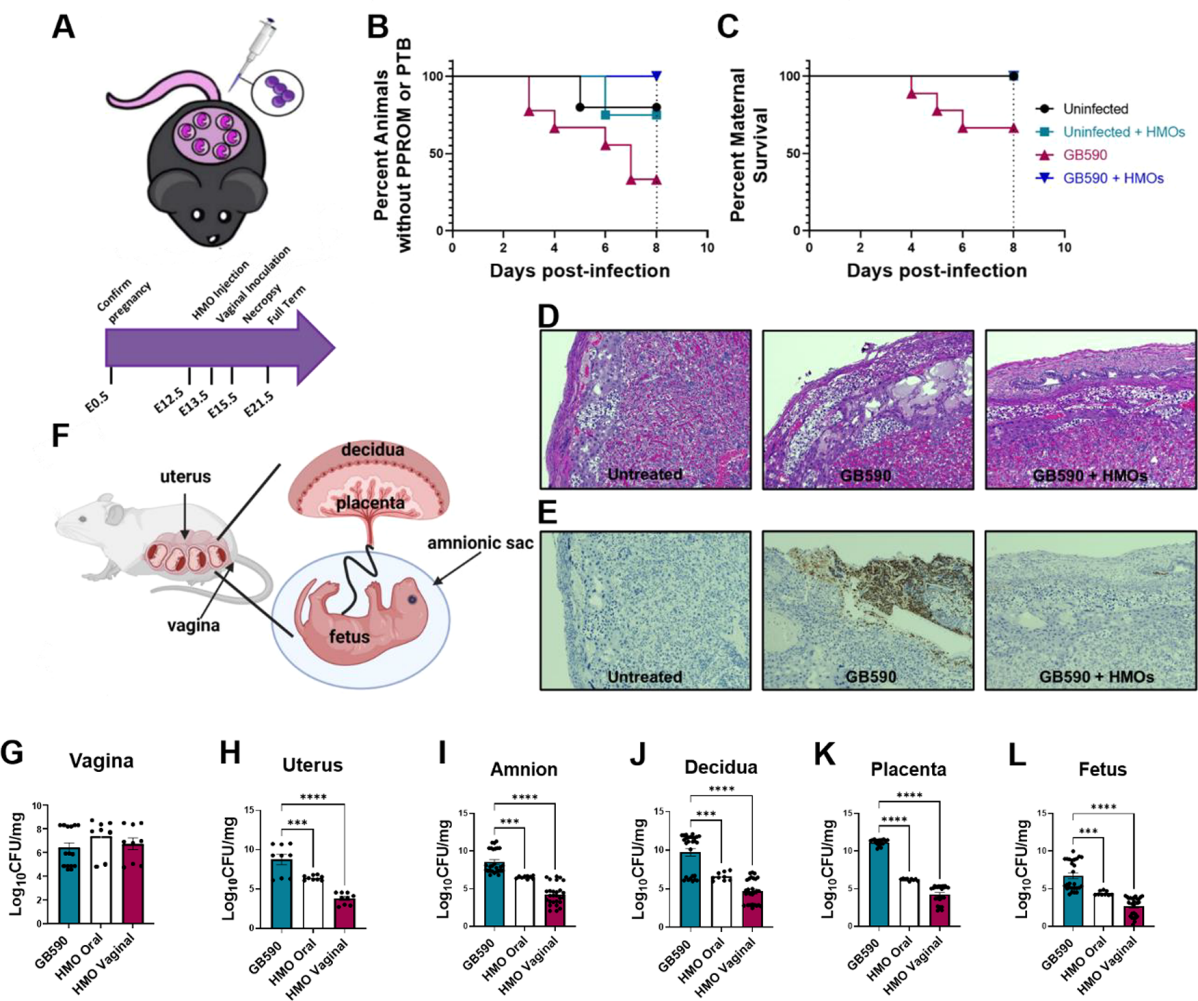
Human milk oligosaccharides reduce cognate disease consequences associated with infection and prevent ascending infection in preclinical mouse model. (A) Conceptual diagram of methods used in these studies. Pregnant mice were treated with HMOs on embryonic day 12.5 (E12.5) and infected with GBS (GB00590) on E13.5. Mice were either sacrificed 2 days postinfection and reproductive tissues were collected for analyses or monitored until E21.5 for adverse disease outcomes associated with pregnancy. Analyses of percent animals (B) without PPROM or preterm birth (PTB) or (C) maternal survival. Dotted line indicates term for the average gestation in the C57BL6/J mouse model used in this study. *p* = 0.0163 Mantel-Cox log-rank test, *n* = 4–9 animals per group with 2 to 3 fetal placental units analyzed per dam (5 uninfected controls, 4 uninfected + HMO controls, and 9 for each of the GBS infected groups). (D) Histopathological examination of the hematoxylin and eosin-stained placental units at 10× magnification. (E) Placental units were analyzed by immunohistochemical techniques using a polyclonal antibody to stain specifically for GBS (indicated by the brown stain). Microscopic imaging is of reproductive tissues from pregnant mice infected with WT GBS (GB590), either with or without 5 mg/mL HMO supplementation. Uninfected controls were also maintained. (F) Conceptual model of reproductive tissues collected and analyzed for GBS burden. Bacterial burden within reproductive tissues was evaluated by quantitative culture, and the HMO cocktail (oral and vaginal injection) promotes significant inhibition with respect to burden within the (H) uterus, (I) amnion, (J) decidua, (K) fetal membranes, and (L) fetus. No significant difference in growth was observed in the (G) vagina. Bars indicate mean ± SEM, * < 0.05 **P < 0.01, ***P < 0.001, ****P < 0.0001 by one-way ANOVA with Tukey’s posthoc multiple comparisons test comparing the addition of the HMO cocktail (oral and vaginal injection) to medium alone controls. Panel F created with Biorender.com.

With the knowledge that a pooled mixture of HMOs prevents GBS adherence to human gestational membranes and EpiVaginal tissue, we sought to determine their role in adverse pregnancy outcomes associated with disease progression. To test this, we intravaginally inoculated pregnant mice with HMOs (5 mg/mL) on embryonic day 14.5 (E14.5) (Figure 2A). As previously described, on E15.5 we infected the pregnant dams with GBS at an infectious dose of 5 × 10^3^ to 1 × 10^4^ CFU allowing us to monitor disease outcomes including PPROM, preterm birth, and maternal death.^23−25^ Uninfected and untreated controls were also maintained. PPROM was recognized by the presence of blood in or surrounding the vagina, and preterm birth was identified by the presence of pups or pup remains, both occurring before E.21.5, the average mouse gestational period. Infection with GB590 resulted in a significant increase in PPROM, preterm birth, and maternal mortality (Figure 2B–C) compared to our untreated population, which was verified by the Mantel-Cox log-rank test (*p* = 0.0163, *p* = 0.079) and Gehan-Breslow-Wilcoxon test (*p* = 0.018, *p* = 0.0803). Our GBS-infected, HMO-treated population experienced no instances of PPROM, preterm birth, or maternal death.

### HMOs Prevent Ascending GBS Infection in a Pregnant Mouse Model

In order to recapitulate perinatal infections of the reproductive tract, we utilized our murine animal model of vaginal colonization and ascending infection of GBS during pregnancy. We have previously shown that GBS colonizes the vaginal mucosa and infiltrates the reproductive tissues of the pregnant mouse.^23,26^ Just as in our disease outcomes model, mice were treated with HMOs (5 mg/mL) on E14.5, followed by either vaginal or oral inoculation of GBS (GB590) on E15.5 at an infectious dose of 5 × 10^2^ to 1 × 10^3^ CFU. Uninfected and untreated controls were also maintained. The mice were sacrificed 48 h postinfection, and necropsy was performed on reproductive tissues (vagina, uterus, decidua, placenta, amnion, fetus) and analyzed for bacterial burden (Figure 2A,F). Surprisingly, we did not detect a difference in bacterial burden in the vagina among our untreated and HMO-treated mice (Figure 2G). While the mice are inoculated vaginally, due to the length of the pipet tip and the burst of the injection, the bacteria often bypass the vagina and initiate infection directly in the cervix. We hypothesize this is the primary reason we do not see significant differences in CFUs among treatment groups. Our HMO-treated population exhibited between a 4- and 7-log decrease in bacterial burden in the uterus, decidua, placenta, amnion, and fetus when administered vaginally (Figure 2H–L), and between a 2- and 5-log decreased when administered orally (Figure 2H–L) when compared to the untreated animals. Additionally, we did not observe bacterial circulation in maternal blood in either of our GBS-infected populations.

### HMOs Reduce Inflammation and GBS Invasion of Reproductive Tissues in a Pregnant Mouse Model

We have previously shown that GBS causes placental inflammation when it invades the gravid reproductive tract in a murine model of infection.^23,24^ To examine the extent of bacterial dissemination and inflammation, reproductive tissues were analyzed by histopathological and immunohistochemical (IHC) techniques. The GB590-infected tissues exhibited increased inflammation as indicated by polymorphonuclear cell infiltration and loss of tissue integrity within the fetal-placental units when compared to the uninfected animals (Figure 2D). The tissues collected from the HMO-treated animals displayed a phenotype similar to that of the uninfected cohort with decreased polymorphonuclear cell infiltration and tissue architecture disruption when compared to the untreated animals. IHC staining with an anti-GBS lysate confirmed increased GBS invasion within the placenta and decidua in the GB590-infected animals when compared to both the HMO-treated and uninfected groups (Figure 2E).

### HMO Supplementation Reverses Proinflammatory Cytokine Response to GBS Infection

**Figure 3:**
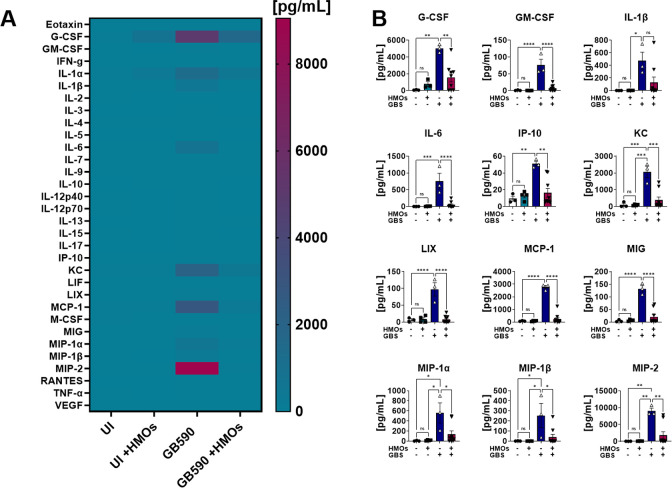
Analysis of cytokine production in decidua tissue in response to GBS infection. (A) Heat map results (teal = lower expression, magenta = higher expression, and cytokine expression concentrations from 0 to 8000 pg/mL) of multiplex cytokine analyses of gestational tissues after ascending vaginal infection with wild-type GB590 (GB590) either with or without 5 mg/mL HMOs, as well as uninfected controls (UI) either with or without 5 mg/mL HMOs. Decidua tissues were collected from pregnant mice on embryonic day E15.5, 2 days post- vaginal infection with GBS. (B) Quantification of G-CSF, GM-CSF, IL-1β, IL-6, IP-10, KC, LIX, MCP-1, MIG, MIP-1α, MIP-1β, and MIP-2 levels revealed that wild-type GB590 infection (dark blue bars) significantly enhances production of these cytokines compared to uninfected controls (white bars); however, HMO supplementation (magenta bars) is significantly reduced in its ability to induce these cytokines compared to the untreated parental strain. Additionally, HMO supplementation in the uninfected controls (teal bars) is often statistically indistinguishable from the uninfected controls (NS). Bars indicate mean values ± standard error mean with individual data points representing results from decidua tissues from individual dams, *n* = 3–9 per group. *P < 0.05, **P < 0.01, ***P < 0.001, ****P < 0.0001, with either one-way ANOVA with Tukey’s posthoc multiple comparisons test. NS = not statistically significant. Results indicate that HMO supplementation reverses the full initiation of proinflammatory cytokine response in decidua tissues in an ascending model of infection during pregnancy.

**Figure 4:**
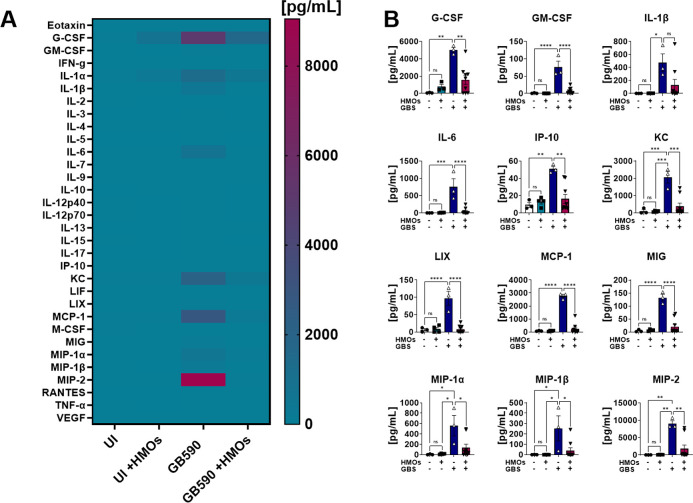
Analysis of cytokine production in placenta tissue in response to GBS infection. (A) Heat map results (teal = lower expression, magenta = higher expression, and cytokine expression concentrations from 0 to 7500 pg/mL) of multiplex cytokine analyses of gestational tissues after ascending vaginal infection with wild-type GB590 (GB590) either with or without 5 mg/mL HMOs, as well as uninfected controls (UI) either with or without 5 mg/mL HMOs. Placenta tissues were collected from pregnant mice on embryonic day E15.5, 2 days postvaginal infection with GBS. (B) Quantification of eotaxin, G-CSF, GM-CSF, IL-1β, IL-6, IL-17, IP-10, LIF, LIX, MCP-1, MIG, and MIP-2 levels revealed that wild-type GB590 infection (dark blue bars) significantly enhances production of these cytokines compared to uninfected controls (white bars); however, HMO supplementation (magenta bars) is significantly reduced in its ability to induce these cytokines compared to the untreated parental strain. Additionally, HMO supplementation in the uninfected controls (teal bars) is often statistically indistinguishable from the uninfected controls (NS). Bars indicate mean values ± standard error mean with individual data points representing results from placental tissues from individual dams *n* = 3–9 per group. *P < 0.05, **P < 0.01, ***P < 0.001, ****P < 0.0001, by one-way ANOVA with Tukey’s posthoc multiple comparisons test. Individual data points represent at least three biological replicates. NS = not statistically significant. Results indicate that HMO supplementation reverses the full initiation of a proinflammatory cytokine response in placental tissues in an ascending model of infection during pregnancy.

**Figure 5:**
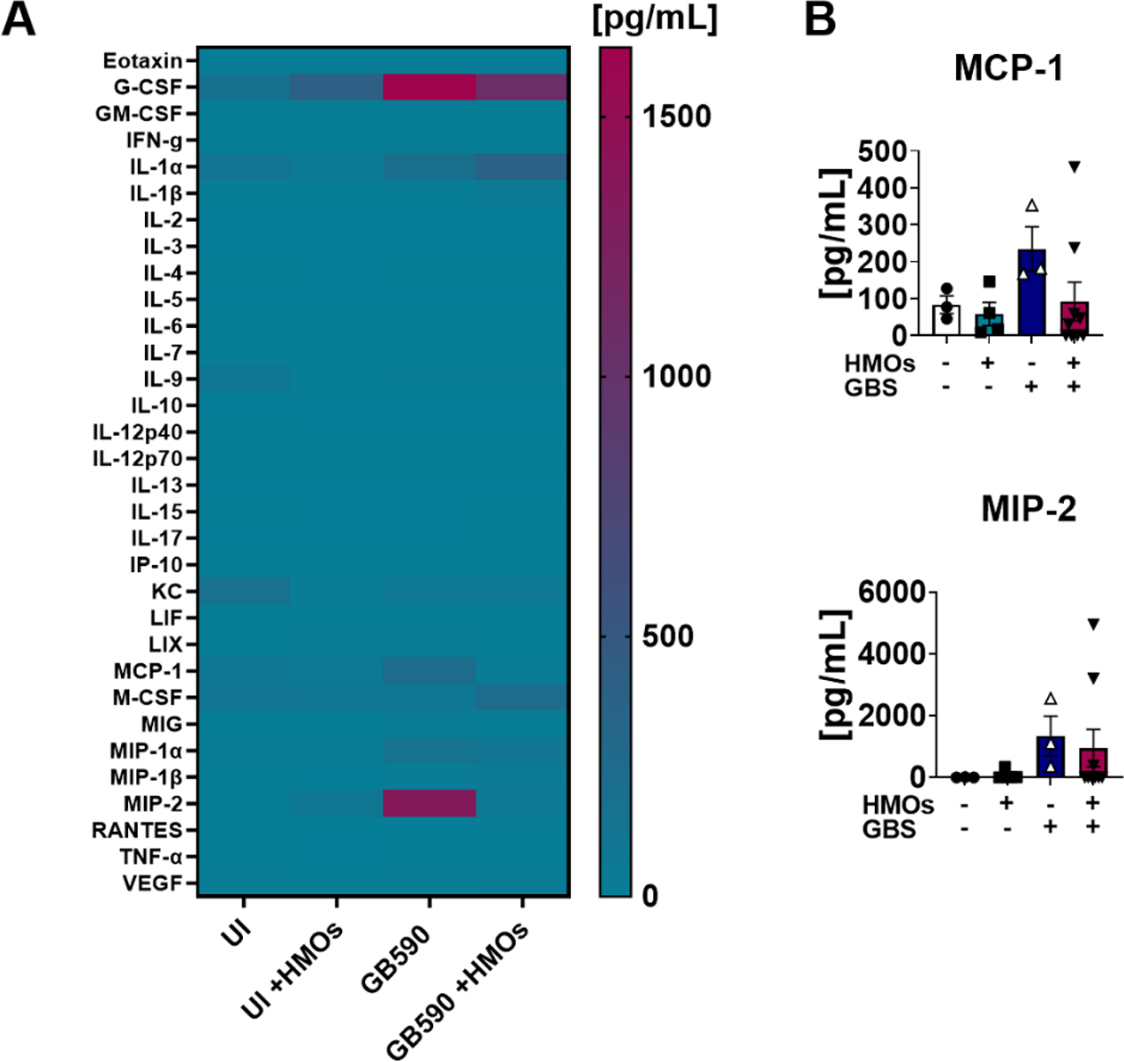
Analysis of cytokine production in amnion tissue in response to GBS infection. (A) Heat map results (teal = lower expression, magenta = higher expression, and cytokine expression concentrations from 0 to 1500 pg/mL) of multiplex cytokine analyses of gestational tissues after ascending vaginal infection with wild-type GB590 (GB590) either with or without 5 mg/mL HMOs, as well as uninfected controls (UI) either with or without 5 mg/mL HMOs. Amnion tissues were collected from pregnant mice on embryonic day E15.5, 2 days postvaginal infection with GBS. (B) Quantification MCP-1 and MIP-2 levels revealed that wild-type GB590 infection (dark blue bars) enhances production of these cytokines compared to uninfected controls (white bars); however, HMO supplementation (magenta bars) is reduced in its ability to induce these cytokines compared to the untreated parental strain. Additionally, HMO supplementation in the uninfected controls teal bars) is often statistically indistinguishable from the uninfected controls (NS). Bars indicate mean values ± standard error mean with individual data points representing results from amnion tissues from individual dams, *n* = 3–9 per group. One-way ANOVA with Tukey’s posthoc multiple comparisons test was performed. Individual data points represent at least three biological replicates. Results indicate that HMO supplementation reverses the full initiation of the proinflammatory cytokine response in amnion tissues in an ascending model of infection during pregnancy.

**Figure 6:**
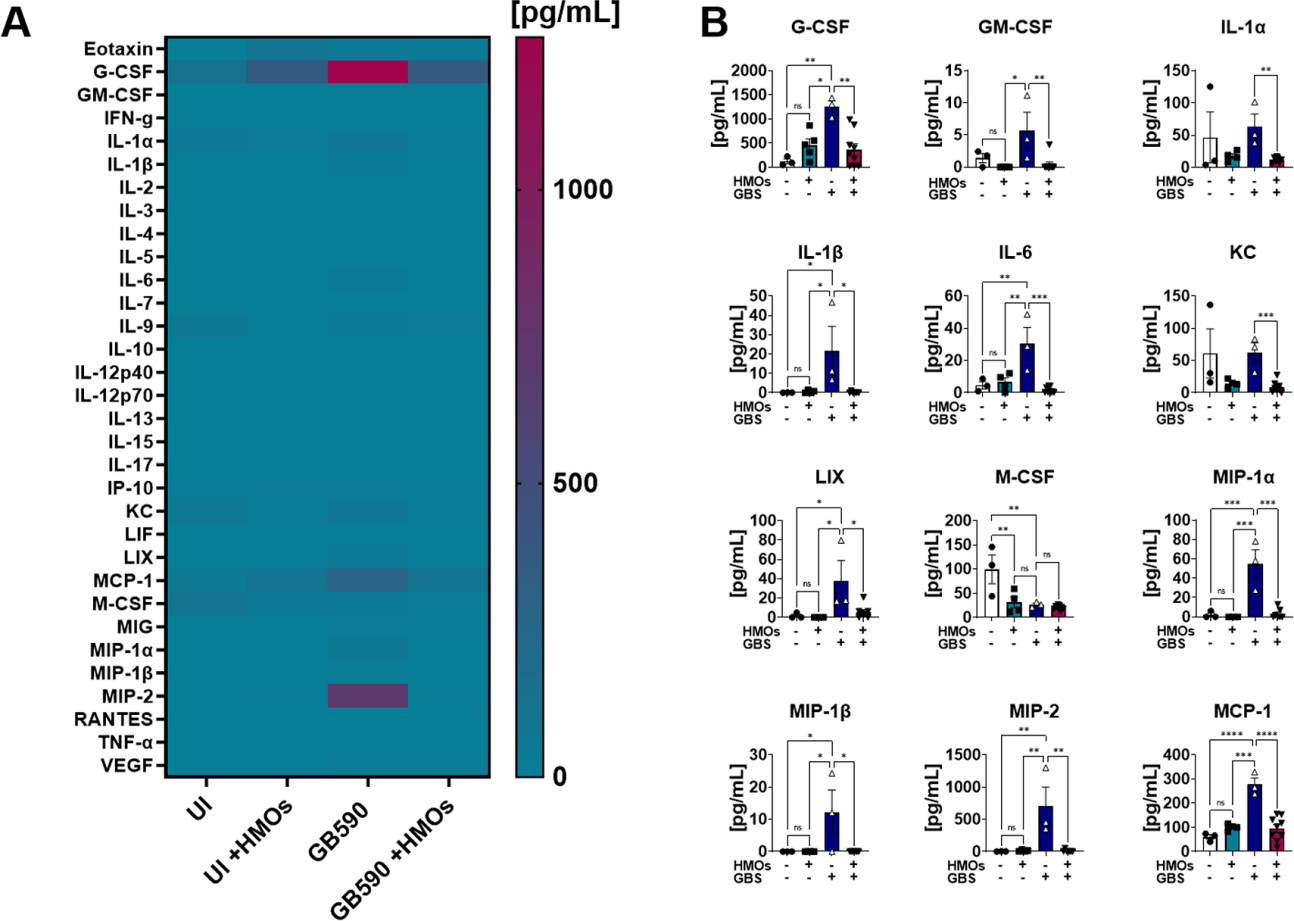
Analysis of cytokine production in fetus tissue in response to GBS infection. (A) Heat map results (teal = lower expression, magenta = higher expression, and cytokine expression concentrations from 0 to 1250 pg/mL) of multiplex cytokine analyses of gestational tissues after ascending vaginal infection with wild-type GB590 (GB590) either with or without 5 mg/mL HMOs, as well as uninfected controls (UI) either with or without 5 mg/mL HMOs. Fetal tissues were collected from pregnant mice on embryonic day E15.5, 2 days postvaginal infection with GBS. (B) Quantification G-CSF, GM-CSF, IL-1α, IL-1β, IL-6, KC, LIX, M-CSF, MIP-1α, MIP-1β, MIP-2, and MCP-1 levels revealed that wild-type GB590 infection (dark blue bars) significantly enhances production of these cytokines compared to uninfected controls (white bars); however, HMO supplementation (magenta bars) is significantly reduced in its ability to induce these cytokines compared to the untreated parental strain. Additionally, HMO supplementation in the uninfected controls (teal bars) is often statistically indistinguishable from the uninfected controls (NS). Bars indicate mean values ± standard error mean with individual data points representing results from fetal tissues from individual dams *n* = 3–9 per group. *P < 0.05, **P < 0.01, ***P < 0.001, ****P < 0.0001, by one-way ANOVA with Tukey’s posthoc multiple comparisons test. Individual data points represent at least three biological replicates. NS = not statistically significant. Results indicate that HMO supplementation reverses the full initiation of the proinflammatory cytokine response in fetal tissues in an ascending model of infection during pregnancy.

Since we have shown the HMO cocktail displays reduced bacterial burden and inflammation of the reproductive tissues in the pregnant mouse model of ascending infection, we hypothesized that this could be associated with alterations in proinflammatory cytokine production as a result of perinatal disease. To interrogate this, we used multiplex cytokine assays to reveal the array of cytokines and chemokines generated within the murine reproductive tissues in response to ascending GBS infection and compared them to those of uninfected controls and HMO supplemented tissues. Our results show that animals infected with WT GB590 exhibited significantly increased production of several proinflammatory cytokines and chemokines in the decidua, placenta, amnion, and fetus (Figures 3, 4, 5, and 6, Figures S4–S8, and Table S3) when compared to uninfected controls (statistical analyses for all cytokine and chemokine analyses include outlier’s test, one way ANOVA, and Student’s *t* test). Most notably, HMO supplementation significantly impairs proinflammatory cytokine and chemokine production, restoring levels comparatively to those of the WT-infected animals. Additionally, HMO supplementation does not alter the production of these cytokines and chemokines in the uninfected animals.

MCP-1 and MIP-2 were upregulated in all reproductive tissues analyzed in the WT GB590-infected mice compared with uninfected controls, and HMO supplementation resulted in a significant loss of proinflammatory cytokine and chemokine production. Similarly, with WT GBS infection, we observed that IL-1β, IL-6, and LIX were enhanced in the decidua, placenta, and fetus (but not the amnion) when compared to uninfected animals, and a significant reduction in production of these cytokines and chemokines was observed with the HMO cocktail compared to that with WT-infected samples. IP-10 and MIG were upregulated in the decidua and placenta in response to bacterial infection compared to uninfected controls, and HMO supplementation caused significant reduction in production of the cytokines and chemokines. KC and MIP-1α were enhanced in the decidua and fetus of WT GBS-infected animals compared to uninfected animals, while the HMO cocktail reversed this increased production of these cytokines and chemokines.

We observed the general trend with significantly increased production of a selection of proinflammatory cytokines and chemokines including G-CSF, GM-CSF, IL-1β, IL-6, IP-10, KC, LIX, MCP-1, MIG, MIP-1α, MIP-1β, and MIP-2 in the decidua (Figure 3 and Figure S4); eotaxin, G-CSF, GM-CSF, IL-1β, IL-6, IL-17, IP-10, LIF, LIX, MCP-1, MIG, and MIP-2 in the placenta (Figure 4 and Figure S5); MCP-1 and MIP-2 in the amnion (Figure 5 and Figure S6); and G-CSF, GM-CSF, IL-1α, IL-1β, IL-6, KC, LIX, M-CSF, MIP-1α, MIP-1β, MIP-2, and MCP-1 in the fetus (Figure 6 and Figure S7) compared to uninfected controls. HMO supplementation reverses enhanced proinflammatory cytokine and chemokine production compared with the GBS-infected animals.

### HMO Exposure Results in Changes in GBS Gene Expression

**Figure 7:**
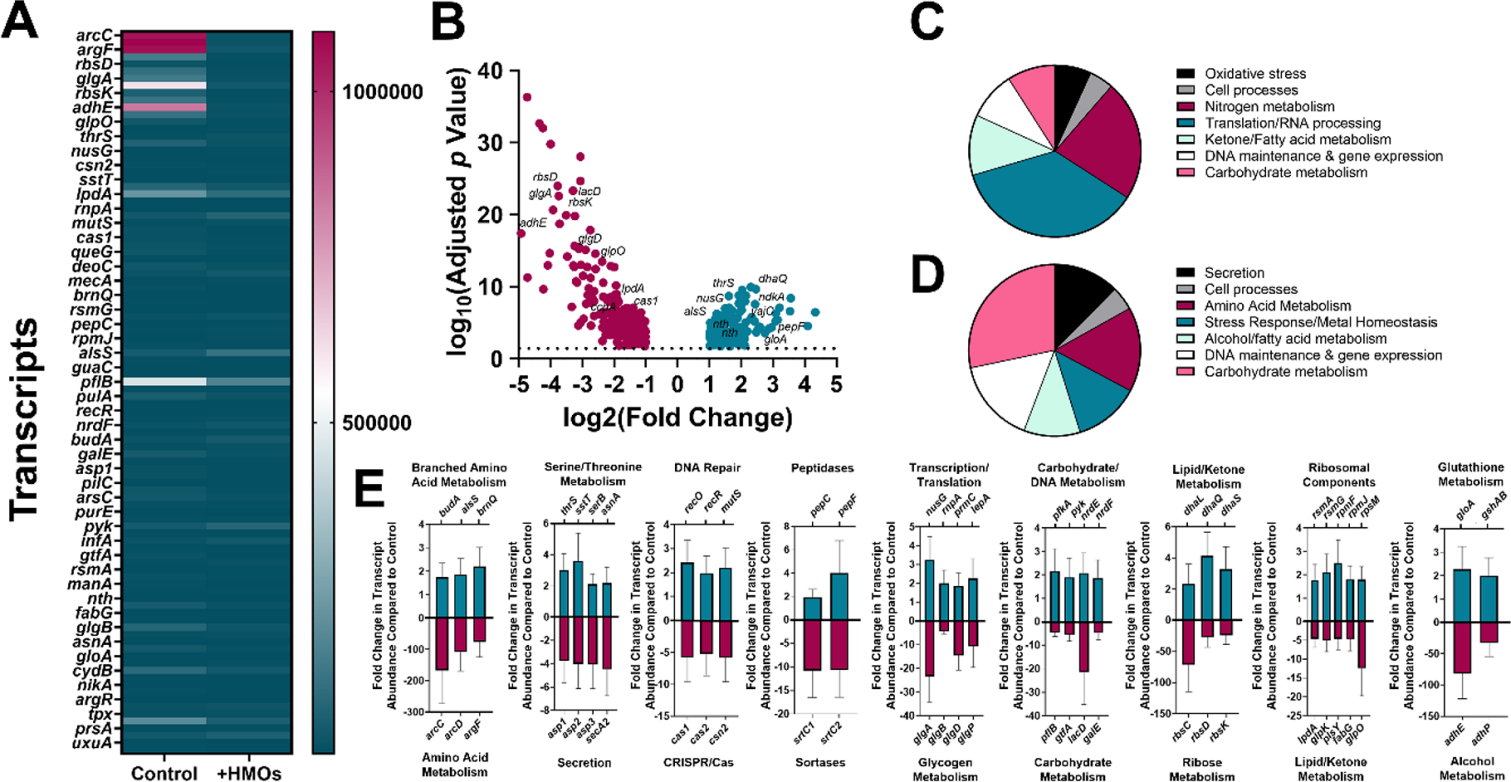
Transcriptomic analysis of differential gene expression (by functional COG analysis) in response to GBS exposure to HMOs. RNA-seq analysis of transcript abundance in GBS grown in medium alone (control) versus medium supplemented with HMOs (+ HMOs) reveals changes in transcript abundance. (A) Heat map of 100 annotated genes differentially expressed in GBS in response to HMO exposure (teal = lower transcript abundance, magenta = higher transcript abundance from 0 to 1 × 10^6^ transcript reads). (B) Volcano plot of downregulated genes (maroon) versus upregulated genes (teal green) in GBS in response to HMO exposure. (C) Pie graph of putative pathways implicated in the genes upregulated in response to HMO exposure, and (D) down-regulated in response to HMO exposure. (E) Specific genes of interest in GBS that were significantly upregulated (teal green) or down-regulated (maroon) in response to HMO exposure (fold change compared to medium alone controls, *P* < 0.05, one-way ANOVA).

To identify GBS responses to HMOs, RNA-seq analyses were employed (Figure 7). Functional classification by COG designation determined that over 400 genes were differentially regulated in response to HMO exposure (Figure S9) including 100 annotated genes (Figure 7A) associated with a variety of cellular functions. HMO exposure resulted in a pronounced reduction in the expression of genes involved with carbohydrate metabolism, ornithine and arginine metabolism, secretion, and glycogen metabolism (Figure 7). Additionally, HMO exposure induced expression of genes involved in DNA repair, RNA synthesis and transcription, serine, threonine, and branched amino acid metabolism and glutathione metabolism (Figure 7). STRING analysis of potential protein–protein interactions among the differentially expressed genes between cells grown in medium alone versus cells grown in the presence of HMOs revealed that GBS gene expression in medium alone is highly enriched for networks associated with carbohydrate (specifically glucose) metabolism (Figure S10A, Table S4). However, in the presence of HMOs, gene expression shifts toward networks enriched for nitrogen metabolism (Figure S10B, Table S4).

## Discussion

Considering recent global health events, vaccine hesitancy is an ever-growing concept. Predating the conceptualization of vaccine and vaccination as medical terminology, this phenomenon of reluctancy in trust of Western medicine has plagued the healthcare system for centuries. While specific to vaccination, new terminology has recently emerged to address the broader resistance of modern healthcare practices. The concept of intervention hesitancy, coined by Rosenthal et al. in early 2022 “address(es) a broad spectrum of disease prevention interventions...beyond vaccine hesitancy.”^27^ While the official name of this idea is new, the principles behind intervention hesitancy have been at play for years and in the context of childbirth preferences. In a 2020 study conducted by Thayer and collogues, maternity care preferences were assessed among U.S. women.^28^ Here they found that over 30% of surveyed women had a novel preference for community care centered birth plans (i.e., midwife facilitated, home births, etc.) compared to hospital births. While this study was small and limited in the diversity of persons surveyed, it highlights an interesting dynamic between doctors and pregnant people in the delivery room.

The collective goal of researchers and public health professionals is the establishment and implementation of safe and effective policies and plans to promote broader public health. In working toward this goal, it has become increasingly imperative to tailor these policies and practices to account for and adapt to changes in public sentiment surrounding medicine. As such, while current efforts for vaccine development, novel antibiotics, and other clinical strategies are vitally important, they are only as effective as their public perception and ultimate utilization. To this end, naturally occurring compounds, such as HMOs may pose an attractive, more palatable alternative to commercial antibiotics as medical intervention techniques. Further, given the global inequity of therapeutic distributions, natural-based compounds may help to close the gap in availability.

HMOs are a group of multifunctional sugars present only in human breast milk. It is well-documented that HMOs function as prebiotics, antiadhesive antimicrobials, and immunomodulators in the infant gut.^29−32^ Based on this known stimulatory activity, our group has previously interrogated how HMOs modulate bacterial growth and biofilm against several pathogenic organisms. Our early research uncovered the impressive bacteriostatic and antibiofilm activity against GBS.^14,15^ Further probing discovered the potent antibiofilm abilities of HMOs against a diverse a bank of clinical isolates of the multidrug resistant, nosocomial pathogen *Acinetobacter baumannii*.^16^ Based on these results, we hypothesized that HMOs could be used to sensitize GBS to antibiotics with limited efficacy. Our group, along with Bode et al., elucidated their powerful adjuvant capabilities with several intracellular-targeting antibiotics against GBS.^33−35^ Through untargeted metabolomic analyses, it was found metabolites affiliated with cell membrane structure and function were perturbed by HMO treatment.^33^ This led us to hypothesize that HMO extracts function by increasing cell membrane permeability. While the culmination of these studies provides promising results, we are continuously seeking to better understand host–pathogen interactions and the HMO-facilitated modulation of those interactions.

GBS has been implicated in perinatal infections since its discovery in the 1930s.^36^ And while it is typically considered a commensal bacterium, it is opportunistic in its ability to cause severe infections in immunocompromised patients. Specifically, neonates are especially at risk, as invasive GBS disease remains a leading cause of infant morbidity and mortality.^37,38^ Even though extensive research has been explored on this pathogen, there is still a lack of understanding of how GBS traverses from the lower genital tract through the extraplacental membrane barrier to cause neonatal infection. We have previously shown in our mouse model of ascending infection during pregnancy that GBS invades the gravid reproductive tract and elicits a proinflammatory response.^26,39,40^

As GBS is an opportunistic pathogen, the prevalence of GBS infections during pregnancy is unsurprising. It is well-known that the instance and severity of infections are increased during pregnancy compared to nonpregnant persons due to the robust immunological changes that occur during this reproductive process.^41^ In the mid 20th century, Sir Peter Medawar made the comparison between the fetus, a semiallograft, and organ transplantation.^42^ He proposed the allograft paradigm to help explain why the fetus, an otherwise foreign body, is not rejected by the maternal immune system. The paternal protein expressing placenta should, under normal immunological conditions, be rejected by the maternal immune system.^41^ The original paradigm relied on the idea of that the maternal immune system is the key modulator of immunologic response to the fetus and various other foreign bodies; however, a modern model suggests integration of the fetal-placental immune response with the maternal immune response.^42^

For a long time, pregnancy was evaluated as a single event, but as science has advanced, we now understand pregnancy as three distinct immune phases. The first phase includes implantation, formation of the placenta, the first trimester, and the early parts of the second trimester. During the first phase, pregnancy resembles that of a wound or infection and as such is considered a proinflammatory phase,^42^ due to an increase in maternal cytokine production. The second phase of pregnancy is marked by a symbiotic relationship between mother and fetus and, thus, the initiation of an anti-inflammatory state within the maternal immune system. Once the fetus has fully developed, the maternal body prepares for delivery by an influx of immune cells to the uterine lining, and a second inflammatory state is initiated. In its totality, pregnancy is marked by both inflammatory and anti-inflammatory immune states, and as such maternal sensitivity to infection varies with gestational stage.^43^

As a common inhabitant of the genitourinary and gastrointestinal tracts, GBS utilizes the vaginal region as a main reservoir for initial colonization. Bacterial transfer from the rectum to the vagina is conventionally accepted as the primary route of passage due to heavy rectal colonization.^44^ Vaginal colonization late in pregnancy is concerning due to the risk of vertical transmission either by ascending transmission *in utero*, during passage through the birth canal, or through aspiration of infected amniotic fluid.^3^ Ascending bacterial infection during pregnancy often leads to excessive inflammation of the gestational membranes surrounding the developing fetus, a condition termed chorioamnionitis. Since vaginal colonization is an important step in the pathogenesis of GBS, we sought to explore mechanisms in which we could prevent GBS adherence to vaginal tissues.

Animal models of vaginal colonization and ascending infection represent useful tools for studying GBS disease during pregnancy. Rodents, and specifically mice, are advantageous due to their low cost, ease of breeding, and well-established models of disease. And while the murine mouse model closely resembles GBS infection of the maternal reproductive tract in humans, there are appreciable differences between the two. Functionally, the uterus houses the developing fetus in both humans and mice; however, anatomically, mice have a bicornuate uterus in which they form two uterine horns, while humans form one single uterine cavity. It is not surprising that the murine vaginal flora contains commensal microbes, but unlike the lactobacilli-dominated microbiota in humans, the more diverse mouse microbiota consists mostly of *Staphylococcus*, *Enterococcus*, and *Lactobacillus* spp.^45^ The reproductive cycle follows a similar progression in both humans and mice, but is much shorter in mice, lasting approximately only 4 days compared to the 28 in humans.^46^

As a result of some of the differences, a vaginal tissue model constructed from human vaginal epithelial cells provides a unique opportunity to study the interactions of GBS with the vaginal epithelium. This 3D tissue model more closely resembles the complexities of the human vagina that has not yet been recapitulated in an animal model of disease. Since we had previously demonstrated GBS colonizes the vaginal mucosa and infiltrates reproductive tissues of the mouse during pregnancy, we hypothesized based on our *in vitro* studies HMOs would prevent or significantly reduce GBS colonization, ascending infection, and adverse pregnancy outcomes associated with disease progression.^23,26,39,40^ Excitingly, treatment with HMOs significantly inhibited GBS adherence to the vaginal epithelium, a result critical toward to the establishment of GBS disease. Patras et al. confirmed HMO treatment also does not impact lactobacilli colonization to human vaginal epithelial cells, underscoring that HMOs might exert targeted effects on pathogens.^47^ Paradoxically, HMO treatment did not alter GBS burden in the mouse vaginal tissues, underscoring the differences between *in vivo* mouse and *ex vivo* human vaginal tissue models. Interestingly, upon interrogation of the inflammatory response in human vaginal organoid tissues utilizing multiplex cytokine analyses, we did not observe significant changes in proinflammatory cytokine production. Of the 16 cytokines analyzed, we observed an increase in IL-1RA with respect to GBS infection, while HMO supplementation reversed this increase (Figure S8). We also observed a similar trend (although not significant) in TNF-α. IL-1RA is a known anti-inflammatory molecule, inhibiting the proinflammatory activity of IL-1; however, it is increasingly appreciated as a critical immunoregulatory molecule.^48^ These results indicate that GBS infection of human vaginal organoids may dampen the vaginal inflammatory response but induce proinflammatory responses in higher reproductive tract tissues, underscoring a tissue compartment-specific response to GBS.

Once GBS colonizes the lower genital tract, this leads to passage into the intrauterine cavity, where the bacterium can cause excessive inflammation of the gestational membranes surrounding the developing fetus. Chorioamnionitis is a major risk factor implicated in PPROM, preterm birth, stillbirth, and neonatal and maternal sepsis.^5,49,50^ In order to bridge the gap between *in vitro* and *in vivo* models of infection, *ex vivo* tissue models can be utilized. *In vitro* models lack the ability to mimic bacterial infections that occur in complex microbial communities and systems, while *in vivo* rodent models do not directly translate to humans. *Ex vivo* tissues isolated from humans allow for experiments with conditions more physiologically relevant to those observed in either *in vitro* or *in vivo* mouse models. In our current studies, we have developed an *ex vivo* model of GBS tissue infection within human extraplacental gestational membranes (EPM).^18,22,51^ Deidentified placental tissues from healthy, term, nonlaboring C-sections were collected, and 12 mm sections were cultured *ex vivo*. We observed that treatment with HMOs significantly reduced GBS adherence and biofilm formation to the EPMs.

We have previously shown GBS invades the gravid reproductive tract and induces host inflammation through enhanced proinflammatory cytokine production in a pregnant mouse model.^39,40^ In our current work, we have demonstrated HMOs are able to reduce bacterial burden and inflammation in the reproductive tissues and fetal compartments when compared to the untreated WT-infected mice. Concurrently, HMO treatment drastically improved maternal survivability and incidences of PPROM and preterm birth as observed in blood surrounding the vagina or the presence of pups or pup remnants in the cage. We hypothesized that this would also correlate with decreased expression of proinflammatory cytokines. We observed a reduction of MCP-1 and MIP-2 in the decidua, placenta, amnion, and fetus. Likewise, IL-1β, IL-6, and LIX were downregulated in the decidua, placenta, and fetus (but not the amnion), while IP-10 and MIG were reduced in the decidua and placenta and KC and MIP-1α were diminished in the decidua and fetus. Reduced bacterial burden, inflammation, and proinflammatory cytokine production were associated with decreased incidences of PPROM, preterm birth, and maternal demise in the HMO-treated, GBS-infected animals when compared to the WT GB590-treated animals. Previous work has correlated enhanced levels of IL-1β, IL-6, MIP-1α, and MIP-2 with a higher risk of PPROM and preterm birth.^52−54^ These studies demonstrate the correlation between bacterial infections such as bacterial vaginosis and UPEC with an upregulation of these proinflammatory markers. Infection leads to an inflammatory response including the release of proinflammatory cytokines and the activation of pathways responsible for cervical ripening and membrane rupture. It is thought that if we can therapeutically inhibit this inflammatory response and the proinflammatory cytokine production, we can limit incidences of preterm birth.^55^

HMO exposure was associated with drastic changes in cellular metabolism, specifically, the switch from expression of genes involved in glucose metabolism to nitrogen metabolism. Similarly, in the related organism *S*. *thermophilus*, alterations in sugar metabolism have been implicated as impacting nitrogen metabolism.^56^ HMO exposure was also associated with the downregulation of several genes associated with GBS virulence, including genes encoding laminin-binding (*lmbA*),^57−59^ sortase activity (*srtA* and *srtB*), and secretion function (*secA2*). Underscoring the important role HMOs can play in altering bacterial virulence gene expression. Additionally, as previously mentioned, untargeted metabolomic analyses uncovered the disruption of several metabolic pathways associated with membrane formation and structural integrity including linoleic acid metabolism, sphingolipid metabolism, glycerophospholipid metabolism, and pyrimidine metabolism.^33^ Transcriptomic analyses corroborated these findings, revealing the upregulation of several genes implicated in ketone and lipid metabolism in response to HMO treatment.

Our group hypothesized the benefits of HMOs go beyond their ability to develop infant microflora by serving as prebiotic and antiadhesive molecules. Our studies demonstrate HMO extracts reduce GBS pathogenesis in our *in vivo* mouse model of ascending vaginal infection during pregnancy. We corroborated these findings through diminished bacterial burden and inflammation of the reproductive tissues and a reduction in proinflammatory cytokine and chemokine production. These results have shown that purified HMOs could be utilized as a potential therapeutic application directed at GBS infections by preventing bacterial colonization and disease progression.

## Data Availability

The raw sequences (FASTQ format) analyzed in this study are available in the NCBI repository under BioProject PRJNA950418 (Biosamples SAMN33991272 to SAMN33991283).

## Supporting Information Available

Raw Expression Data (XLSX)
Comparative Raw Expression Data (XLSX)
Deseq2 (XLSX)
Materials and Methods. Figure S1, MALDI-FT-ICR spectra of 5 donor milk samples used in the study; Figure S2, Analysis of oligosaccharide standards on TOF MS; Figure S3, Human milk oligosaccharides reduce bacterial adherence to gestational membranes; Figure S4, Analysis of cytokine production in decidua tissue in response to GBS infection; Figure S5, Analysis of cytokine production in placenta tissue in response to GBS infection; Figure S6, Analysis of cytokine production in amnion tissue in response to GBS infection; Figure S7, Analysis of cytokine production in fetal tissue in response to GBS infection; Figure S8, Analysis of cytokine production in EpiVaginal tissues in response to GBS infection; Figure S9, Heatmap of 474 significant transcripts identified with DESeq2; Figure S10, STRING analysis of potential protein–protein interactions in GBS in the presence or absence of HMOs; Table S1, Group B *Streptococcus* strains evaluated in the study; Table S2, Generic structural descriptions of the molecular ions observed during analysis of HMOs in human milk samples; Table S3, Comparison of cytokines displaying a phenotype in response to HMO treatment; Table S4, STRING analyses of predicted protein–protein-interactions (PDF)
