# Supplementary material for: The Utility of Human Milk Oligosaccharides against Group B Streptococcus Infections of Reproductive Tissues and Cognate Adverse Pregnancy Outcomes
Source: ACS Cent Sci. 2023 Aug 9;9(9):1737–49. doi: 10.1021/acscentsci.3c00101 (PMC10540283; doi:10.1021/acscentsci.3c00101)
Supplement: Supplementary file 4 — oc3c00101_si_005.pdf [file oc3c00101_si_005.pdf]

Supporting Information

The utility of human milk oligosaccharides against Group B *Streptococcus* infections of reproductive tissues and cognate adverse pregnancy outcomes

Rebecca E. Moore[1,2], Sabrina K. Spicer[3], Jacky Lu[4], Schuyler A. Chambers[5], Kristen N. Noble[6], Jonathan Lochner[6], Rebecca C. Christofferson[7], Karla A. Vasco[8], Shannon D. Manning[8], Steven D. Townsend[3*], Jennifer A. Gaddy[1,2,4*]

[1]Department of Medicine, Vanderbilt University Medical Center, Nashville, Tennessee, U.S.A.

[2]Department of Veterans Affairs, Tennessee Valley Healthcare Systems, Nashville, Tennessee, U.S.A.

[3]Department of Chemistry, Vanderbilt University, Nashville, Tennessee, U.S.A.

[4]Department of Pathology, Microbiology and Immunology, Vanderbilt University Medical Center, Nashville, Tennessee

[5]Department of Chemistry, Stanford University, Stanford, California, U.S.A.

[6]Department of Pediatrics, Vanderbilt University Medical Center, Nashville, Tennessee, U.S.A.

[7]Department of Pathobiological Sciences, School of Veterinary Medicine, Louisiana State University, Baton Rouge, Louisiana, U.S.A.

[8]Michigan State University, Department of Microbiology and Molecular Genetics, East Lansing, Michigan, U.S.A.

*Lead Contacts (denotes co-corresponding authorship)

Email: jennifer.a.gaddy@vumc.org

Email: steven.d.townsend@vanderbilt.edu

Table of Contents

Materials and Methods……S4

- Safety Statement……S4
- Bacterial Strains and Culture Conditions……S4
- HMO Isolation……S4
- MS and MS/MS Analysis of HMO Samples……S5
- Epi Vaginal Coculture……S5
- Gestational Membrane Coculture……S6
- Quantifying Bacterial Adherence in Gestational Membranes……S6
- High-Resolution Field-Emission Gun Scanning Electron Microscopy (FEG-SEM) Analyses……S7
- Mouse Model of Ascending Vaginal GBS infection During Pregnancy……S7
- PPROM, Preterm Birth, and Survival Analyses……S8
- Quantifying Bacterial Burden in Host Tissues……S8
- Histopathological Analyses……S8
- Immunohistochemical Analyses……S9
- Cytokine Analyses……S9
- RNA Extraction and Transcriptomic Analyses……S9
- STRING Analyses……S10
- Statistical Analyses……S10
- Ethics Statement. ……S11

Supplemental Tables and Figures……S12

- Table S1. Group B *Streptococcus* strains evaluated in the study……S12
- Figure S1. MALDI-FT-ICR spectra of 5 donor milk samples used in the study……S12
- Figure S2. Analysis of oligosaccharide standards on TOF MS……S13
- Table S2. Generic structural descriptions of the molecular ions observed during analysis of HMOs in human milk samples……S14
- Figure S3. Human milk oligosaccharides reduce bacterial adherence to gestational membranes……S14
- Figure S4. Analysis of cytokine production in decidua tissue in response to GBS infection……S15

Figure S5. Analysis of cytokine production in placenta tissue in response to GBS infection……………………………………………………………………………………………S16

Figure S6. Analysis of cytokine production in amnion tissue in response to GBS infection……………………………………………………………………………………………S17

Figure S7. Analysis of cytokine production in fetal tissue in response to GBS infection….S18

Figure S8. Analysis of cytokine production in EpiVaginal™ tissues in response to GBS infection……………………………………………………………………………………………S19

Table S3. Comparison of cytokines displaying a phenotype in response to HMO treatment…………………………………………………………………………………………...S20

Figure S9. Heatmap of 474 significant transcripts identified with DESeq2…………………S21

Figure S10. STRING analysis of potential protein-protein interactions in GBS in the presence or absence of HMOs………………………………………………………………..S22

Table S4. STRING analyses of predicted protein-protein-interactions……………………...S22

References……………………………………………………………………………………S23

Materials and Methods

Safety Statement

No unexpected or unusually high safety hazards were encountered in this study.

Bacterial Strains and Culture Conditions

The bacterial strains used in this study are shown in Table S1. All bacterial strains were grown on tryptic soy agar plates supplemented with 5% sheep blood (blood agar) plates at 37˚C in ambient air overnight. Bacteria were sub-cultured from blood agar plates into Todd-Hewitt broth (THB) and incubated at 37°C in ambient air overnight. The following day, bacterial density was measured spectrophotometrically at an optical density of 600 nm (OD_{600}), and bacterial numbers were determined with a coefficient of 1 OD_{600} = 10^9 CFU/mL.

HMO isolation

Human milk was obtained from healthy, lactating women between 3 days and 3 months postpartum and stored between −80 and –20°C. Deidentified milk was provided by Dr. Jörn-Hendrik Weitkamp from the Vanderbilt Department of Pediatrics, under a collection protocol approved by the Vanderbilt University institutional review board (IRB #100897), or from Medolac. Milk samples were thawed and then centrifuged for 45 min. Following centrifugation, the resultant top lipid layer was removed. The proteins were then removed by diluting the remaining sample with roughly 1:1 (vol/vol) 180 or 200 proof ethanol, chilling the sample briefly, and centrifuging for 45 min, followed by removal of the resulting HMO-containing supernatant. Following concentration of the supernatant in vacuo, the HMO-containing extract was dissolved in 0.2 M phosphate buffer (pH 6.5) and heated to 37°C. β-Galactosidase from Kluyveromyces lactis was added, and the reaction mixture was stirred until lactose hydrolysis was complete. The reaction mixture was diluted with roughly 1:0.5 (vol/vol) 180 or 200 proof ethanol, chilled briefly, and then centrifuged for 30 min. The supernatant was removed and concentrated in vacuo, and the remaining salts, glucose, and galactose were separated from the oligosaccharides using size

exclusion chromatography with P-2 gel (H2O eluent). The oligosaccharides were then dried by lyophilization. Correspondingly, HMO isolates from donors were combined and solubilized in water to reach a final concentration of 102.6 mg/ml.

MS and MS/MS Analysis of HMO Samples

Dried HMO samples were prepared and processed for evaluation by reconstitution in water to approximately 1 mg/mL. These solutions were deposited on a matrix-assisted laser desorption/ionization (MALDI) target plate as follows: 1 µL of HMO was spotted followed by 0.2 µL of 10 mM NaCl and 1 µL of DHB matrix (60 mg/mL in 50% methanol). The spots were allowed to air-dry and then were analyzed in positive ion mode on a 9.4T Fourier transform ion cyclotron resonance (FT-ICR) mass spectrometer (MS) (Bruker Solarix). Mass spectra were acquired in positive ion mode from m/z 300 to 2500. Sodium ion adducts of HMOs were detected with a mass accuracy of >2 ppm. MS/MS analysis was performed for selected ions with a linear ion trap mass spectrometer equipped with a MALDI source (LTQ XL, Thermo Scientific). Selected sodium adduct ions of interest were isolated with a 1 amu window and fragmented via CID using a collision energy of 35 eV (Supplemental Figure 1-3).[1]

Epi Vaginal Coculture

EpiVaginal™ (VEC-100™) tissues were purchased from MatTek Life Sciences. These tissues are cultured from normal, primary human-derived vaginal epithelial cells, and are similar to studying *in vivo* tissue. The tissues were processed into 8-mm diameter sections, and sections were placed in Nunc™ single well tissue culture plate inserts containing DMEM medium, F12 medium, phenol-red cell culture medium, supplemented with 5 µg/ml gentamicin (10% of normal gentamicin level) and 0.25 µg/ml amphotericin B. Sections were thawed and incubated overnight at 37°C in ambient air containing 5% CO_2; washed 3 times with prewarmed, sterile phosphate buffered saline (pH 7.4); and placed in VEC-100-MM™ (MatTek Life Sciences) maintenance

medium. Bacterial cells were added to the top face of the tissue at a multiplicity of infection of 1 × 10^6 cells per 8-mm diameter membrane, using a predetermined coefficient of bacterial density of 1 OD_{600} = 10^9 CFU/mL. HMOs were added to achieve a final carbohydrate concentration of ca. 5 mg/mL. Concomitantly, uninfected vaginal tissue samples were also maintained. Cocultures were incubated at 37°C in ambient air containing 5% CO_2 for 24 hours and cells were fixed with 2.0% paraformaldehyde and 2.5% glutaraldehyde in 0.05 M sodium cacodylate buffer (Electron Microscopy Sciences, Hatfield, PA) for at least 12 hours prior to processing for microscopy.

Gestational Membrane Coculture

De-identified gestational membrane tissue samples were procured from term, non-laboring Caesarean section-delivery live births at Vanderbilt University Medical Center with approval from the Vanderbilt University Medical Center Institutional Review Board (VUMC IRB #181998). 12-mm gestational membranes biopsy punches were isolated and cultured in RPMI 1640 medium (ThermoFisher, Waltham, MA) with 10% charcoal stripped fetal bovine serum (ThermoFisher) and 1% antibiotic/antimycotic solution (ThermoFisher) overnight at 37°C in room air supplemented with 5% carbon dioxide. The membranes were washed 3 times, infected with 10^6 CFU/mL of GBS in RMPI 1640 medium without antibiotics in the absence of HMO treatment or supplemented with HMOs at a concentration of 5 mg/mL. A predetermined coefficient of bacterial density of 1 OD_{600} = 10^9 CFU/mL. Cocultured tissues were incubated at 37°C in air supplemented with 5% carbon dioxide overnight and cells were fixed with 2.0% paraformaldehyde and 2.5% glutaraldehyde in 0.05 M sodium cacodylate buffer (Electron Microscopy Sciences, Hatfield, PA) for at least 12 h prior to processing for microscopy.

Quantifying Bacterial Adherence in Gestational Membranes

To determine bacterial adherence in gestational membranes, quantitative culture methods were employed. Following overnight incubation at 37°C in air supplemented with 5% carbon

dioxide, gestational membranes were washed three times and placed in mL of sterile THB. Tissues were homogenized and subjected to serial dilution and plating onto blood agar to enumerate bacteria (CFU/mL) in host tissue.

High-Resolution Field-Emission Gun Scanning Electron Microscopy (FEG-SEM) Analyses

Bacterial adherence was analyzed via FEG-SEM as previously described.[2-4] Briefly, bacterial cells were cultured in biofilms adhering to gestational membranes overnight in the culture conditions described above. HMOs were dissolved in DI water to achieve a concentration of 102.6 mg/mL and filtered through a 0.2 µm syringe filter. HMOs were added to achieve a final carbohydrate concentration of ca. 5 mg/mL. The following day, bacterial cells were fixed in a solution of 2.5% glutaraldehyde, 2.0% paraformaldehyde, and 0.05 M sodium cacodylate buffer pH 7.4. Samples were dehydrated with sequential washes of increasing concentrations of ethanol before being subjected to critical point drying, mounting on aluminum stubs, and sputter coating with 20 nm of gold–palladium. Samples were viewed using an FEI Quanta 250 field-emission gun scanning electron microscope at 5 kEV with a spot size of 2.5.

Mouse Model of Ascending Vaginal GBS infection During Pregnancy

GBS infection of pregnant mice and subsequent analyses were performed as previously described.[5, 6] Briefly, C57BL6/J mice were purchased from Jackson laboratories and mated in harem breeding strategies (1 male to 3-4 females) overnight. The following day, pregnancy was confirmed by the presence of a vaginal mucus plug establishing the embryonic date (E0.5). On embryonic day 12.5 (E12.5) pregnant dams were anesthetized via inhalation of isoflurane and vaginally injected or dosed orally with an HMO cocktail at a concentration of ca. 5 mg/mL. On embryonic day 13.5 (E13.5) pregnant dams were anesthetized via inhalation of isoflurane and vaginally infected by pipetting $5x10^2$ to 10^3 colony forming units (CFU) in 0.05 mL of THB plus 10% gelatin directly into the vagina. It is important to note that the release of the liquid might

create a pressure effect where some of the inocula could be propelled into the cervix. Uninfected controls were also maintained. On embryonic day 15.5 (E15.5) animals were euthanized by carbon dioxide asphyxiation and necropsy was performed to harvest reproductive tissues including vagina, uterus, placenta, decidua, fetal membranes, and fetus.

PPROM, Preterm Birth, and Survival Analyses

GBS infection of pregnant mice and subsequent analyses were performed as previously described.[5, 6] Briefly, C57BL6/J mice were purchased from Jackson laboratories and mated in harem breeding strategies (1 male to 3-4 females) overnight. The following day, pregnancy was confirmed by the presence of a vaginal mucus plug establishing the embryonic date (E0.5). On embryonic day 12.5 (E12.5) pregnant dams were anesthetized via inhalation of isoflurane and vaginally injected with an HMO cocktail at a concentration of ca. 5 mg/mL. On embryonic day 13.5 (E13.5) pregnant dams were anesthetized via inhalation of isoflurane and vaginally infected (directly into the cervix) with $5x10^3$ to $1x10^4$ colony forming units (CFU) in 0.05 mL of THB plus 10% gelatin. Uninfected, and infected (untreated) controls were also maintained. Animals were monitored daily for PPROM, preterm birth, and maternal survival. On embryonic day 21.5 (E21.5) animals were euthanized by carbon dioxide asphyxiation.

Quantifying Bacterial Burden in Host Tissues

To determine bacterial burden in reproductive tissues quantitative culture methods were employed as previously described.[5] Briefly, reproductive tissues were weighed and placed in sterile THB (Todd-Hewitt broth which is optimized for growth of streptococcal species). Tissues were homogenized and subjected to serial dilution and plating onto blood agar to enumerate bacteria (CFU/mg) in host tissue.

Histopathological Analyses

Reproductive tissues were subjected to a primary fixation in 4% formalin (neutral buffered) overnight. The following day, tissues were embedded in paraffin and sectioned into 5 m thick sections for staining and microscopical analyses. Sections were stained with hematoxylin and eosin for histopathological examination and imaged with an OMAX M83ES compound light microscope with ToupView software package.

Immunohistochemical Analyses

Tissues were fixed in 4% neutral buffered formaldehyde overnight before being embedded into paraffin blocks. Samples were cut into 5-μm sections, and multiple sections were placed on each slide for analysis. Samples were deparaffinized with xylene, and heat-induced antigen retrieval was performed on the Bond Max automated IHC stainer (Leica Biosystems) using Epitope Retrieval 2 solution for 5 to 20 min. Slides were incubated with a rabbit polyclonal anti-GBS antibody (ab78846; Abcam) for 1 h. The Bond Polymer Refine detection system (Leica Biosystems) was used for visualization. Slides were counter stained with eosin, dehydrated and cleared, and coverslips were added before light microscopy analysis was performed.

Cytokine Analyses

Mouse reproductive tissues, maternal sera, and amniotic fluid were analyzed by multiplex cytokine assays. Mouse tissues were placed in 1 mL of sterile PBS or THB+ 10 mg/mL penicillin and homogenized and passed through a 0.22 μm filter. Samples were frozen at -80˚C or on dry ice until analyses were performed. Samples were analyzed by Eve Technologies via multiplex cytokine array (Eve Technologies, Alberta, Canada) as previously described[77]. We have previously validated host targets for specific cytokines (IL-1β, IL-6, KC, and TNF-α) by sandwich ELISA (AbCam).[7, 8]

RNA Extraction and Transcriptomic Analyses

Transcriptomic analyses were performed as previously described.[8] Briefly, bacteria were cultured in THB broth alone or supplemented with 2.5 mg/mL of HMOs (a sub-inhibitory concentration) at 37˚C for 4 hours. 15 mL cultures were centrifuged at 8,000 x g and the supernatant was removed from the cell pellet. Cells were resuspended in 1 mL of RNAprotect Bacteria reagent (Qiagen) and stored at -80˚C until RNA extraction protocol was performed. RNA was extracted using the RNeasy Mini Kit (Qiagen) with enzymatic lysis, proteinase K treatment, and mechanical disruption. DNA was removed using the Turbo DNA-free kit (Ambion) and ribosomal RNA was removed using the Ribo-Zero rRNA removal kit for Gram-positive bacteria (Epicentre). Libraries were prepared using TruSeq Stranded mRNA library preparation kit (Illumina) and RNAseq was performed by Hudson Alpha using a HiSeq X Ten. Base calling was performed with the Illumina Real Time Analysis (RTA) v1.18.64 and the resulting data was converted to FastQ files with Illumina Bcl2fastq, v1.8.4. Adapter and quality trimming were carried out with Trim Galore! v.0.6.4; while, ribosomal RNA was removed with SortMeRNA v.2.1.[9] The accession information for the reference GBS genome, utilized for aligning the RNA-Seq reads, as well as the locus tag key employed for facilitating cross-identification of annotated transcripts, were obtained from the genome of *Streptococcus agalactiae* strain NEM316, assembly ASM19605v1 (GeneBank assembly GCA_000196055.1). Transcripts were mapped to the published genome of *Streptococcus agalactiae* strain NEM316 using STAR v.2.6.1d and quantified with Salmon v.1.2.1.[10] Differential expression analysis was performed using DESeq2 comparing samples grown in medium supplemented with HMOs to samples grown in medium alone. Genes with at least a 2-fold change in expression and $P<0.05$ as determined by one-way ANOVA were considered to have significant differences in expression.

STRING Analyses[11]

To identify putative protein-protein interactions and networks of changes in gene expression, differentially expressed genes were uploaded to the Search Tool for the Retrieval of Interacting

Genes/Proteins (STRING) database (https://string-db.org/) and significant protein-protein interactions were determined at the criterion of confidence (combined score) > 0.4.

Statistical Analyses

Statistical analysis of parametric data with more than two groups was performed using one-way ANOVA with either Tukey's or Dunnet's *post hoc* correction for multiple comparisons; all reported *P* values were adjusted to account for multiple comparisons. For parametric data with two groups, a Student's *t* test was used. *P* values of ≤0.05 were considered significant. Non-parametric data (such as log-transformed CFU data) were analyzed by Mann-Whitney U or Kruskal-Wallis tests. Effect size (Cohen's D) was calculated from observed differences between the uninfected and infected groups to determine the smallest group size that would enable detection of a significant effect at a minimum desired power of 80%. The power statistics were calculated based on the hypothesis focused on proinflammatory cytokines where an effect was expected; namely, IL-6, IFN-g, and IL-1B. To justify the minimum group size, we focused on the smallest possible effect size among the comparisons of interest, which was IFN-g. Using the 'pwr' package, a minimum effect size of 1.53 was necessary for power of 80% with a group size of n=3. Cohen's D for the comparison of IFNg between groups was 1.67; thus, a minimum group size of 3 individuals is sufficiently powered to detect differences. All data analyzed in this work were derived from at least three biological replicates (representing different placental samples). Statistical analyses were performed using GraphPad Prism 9 (GraphPad Software Inc.) or R version 4.2.1 in R Studio 2022.07.0.6).

Ethics Statement

This study was carried out in accordance with the recommendations of the Vanderbilt University Medical Center Institutional Review Board. This protocol was approved by the Institutional Review Board (IRB #181998 and #00005756). All animal experiments were performed in

accordance with the Animal Welfare Act, U.S. federal law, and NIH guidelines. All experiments were carried out under a protocol approved by Vanderbilt University Institutional Animal Care and Use Committee (IACUC: M/14/034 and M/17/012), a body that has been accredited by the Association of Assessment and Accreditation of Laboratory Animal Care Act (AAALAC).

Supplemental Tables and Figures

Table S1. Group B *Streptococcus* strains evaluated in the study.

Strain ID	Strain Type	Sequence Type	Capsular Serotype	Isolation Source
GB0002	Colonizing	ST-23	CpsIa	Vaginal/rectal colonization
GB0037	Invasive	ST-1	CpsV	EOD/sepsis
GB0112	Colonizing	ST-12	CpsIII	Vaginal/rectal colonization
GB0590	Colonizing	ST-19	CpsIII	Vaginal/rectal colonization
10/84	Invasive	ST-26	CpsV	Blood

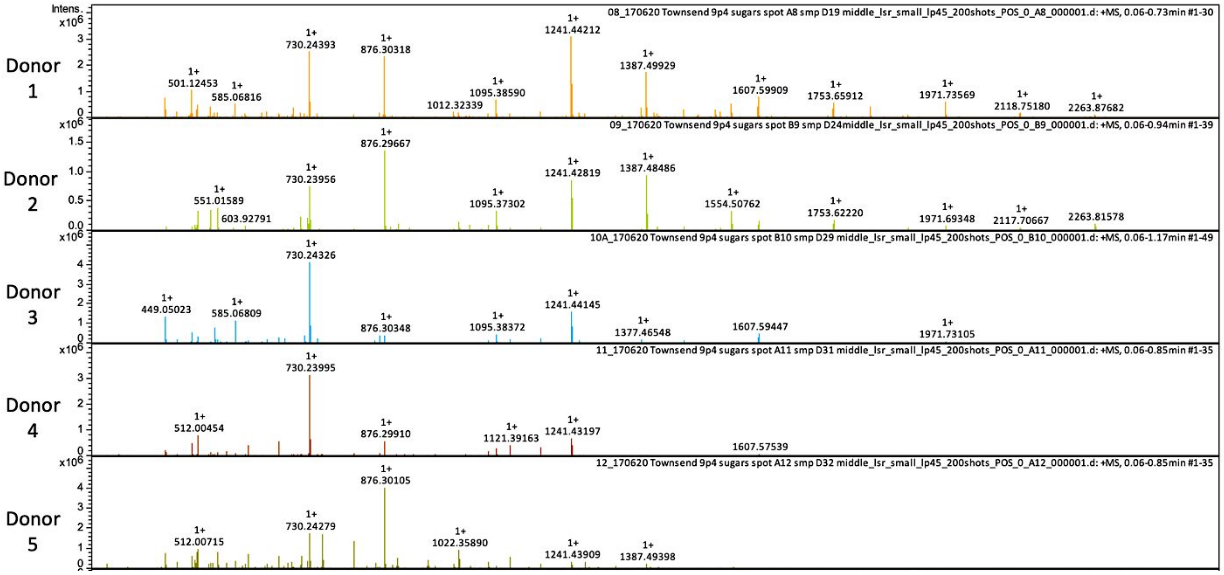

Figure S1. MALDI-FT-ICR spectra of 5 donor milk samples used in the study.

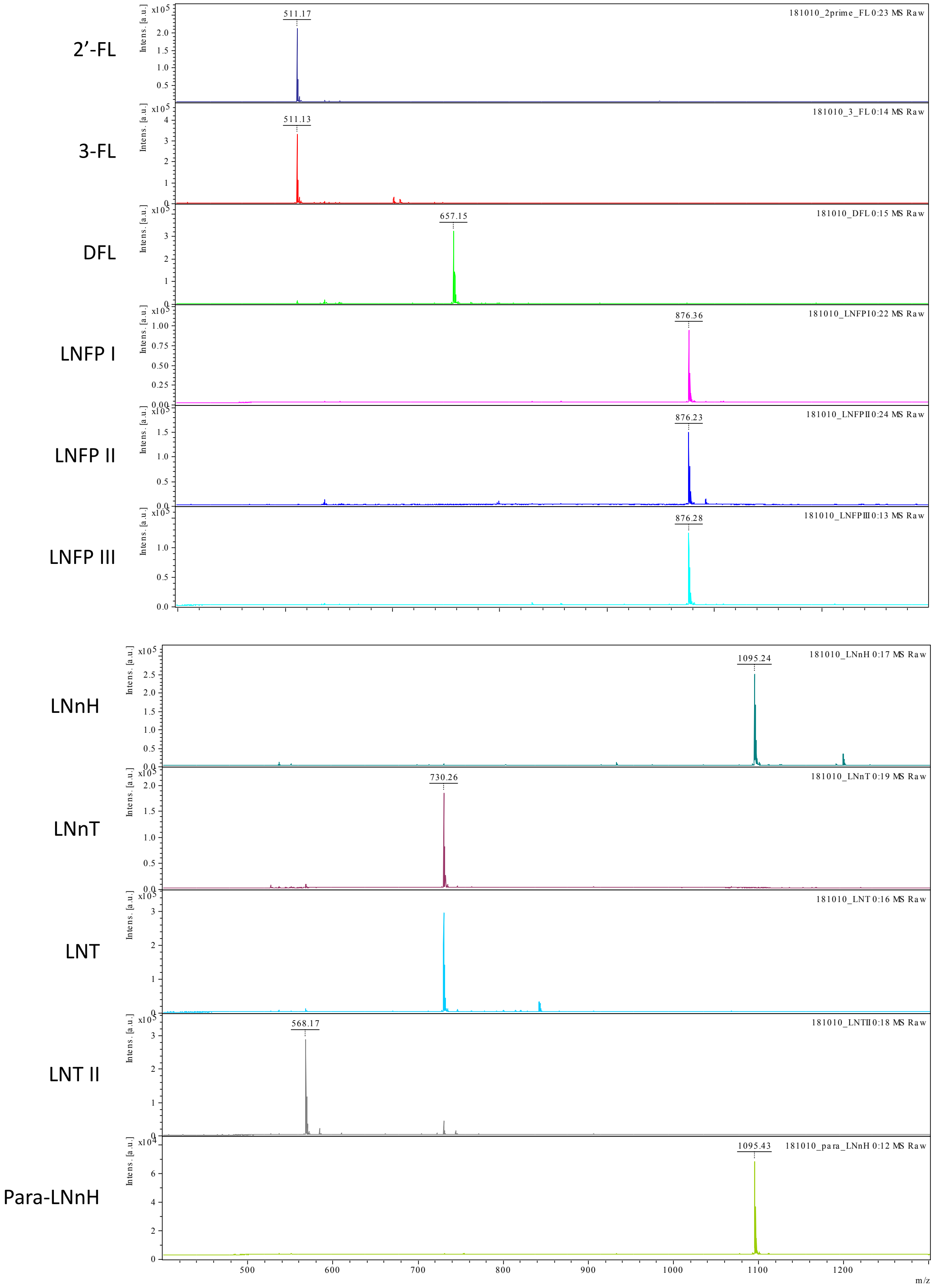

Figure S2. Analysis of oligosaccharide standards on TOF MS. Standards prepared as 1mg/mL in water, spotted on target plate with DHB and Na, and run on Ultrafelx MS in reflectron positive mode. Signals shown are [M+NA+].

Table S2. Generic structural descriptions of the molecular ions observed during analysis of HMOs in human milk samples. The *exact mass* is the mass calculated from a molecular formula using known masses of the most abundant isotopes of each element in the structure. *[M+Na+]* is the calculated mass of the sodium adduct that's expected after ionization. *Measured* is the mass value observed by the instrument. Ppm is a measurement of the error that exists between the expected mass and the observed mass. A negative ppm represents a measured mass that's greater than what was expected. A positive ppm represents a measured mass that's lower than what was expected.

Compound	Exact Mass	[M+Na+]	Measured	ppm
3-fucosyllactose (3-FL)	488.17	511.160	511.130	58.69004
2'-fucosyllactose (2'-FL)	488.17	511.160	511.166	-11.738
Difucosyllactose (DFL)	634.23	657.220	657.149	108.0308
Lacto-N-fucopentaose I (LNFP I)	853.31	876.300	876.359	-67.3285
Lacto-N-fucopentaose II (LNFP II)	853.31	876.300	876.227	83.3048
Lacto-N-fucopentaose III (LNFP III)	853.31	876.300	876.284	18.25859
Lacto-N-triose II (LNT II)	545.20	568.190	568.172	31.67954
Lacto-N-neohexaose (LNnH)	1072.38	1095,370	1095.243	115.9526
para-lacto-N-neohexaose (para-LNnH)	1072.38	1095.370	1095.432	-56.6019
Lacto-N-tetraose (LNT)	707.25	730.240	730.151	121.8777
Lacto-N-neotetraose (LNnT)	707.25	730.240	730.262	-30.1271

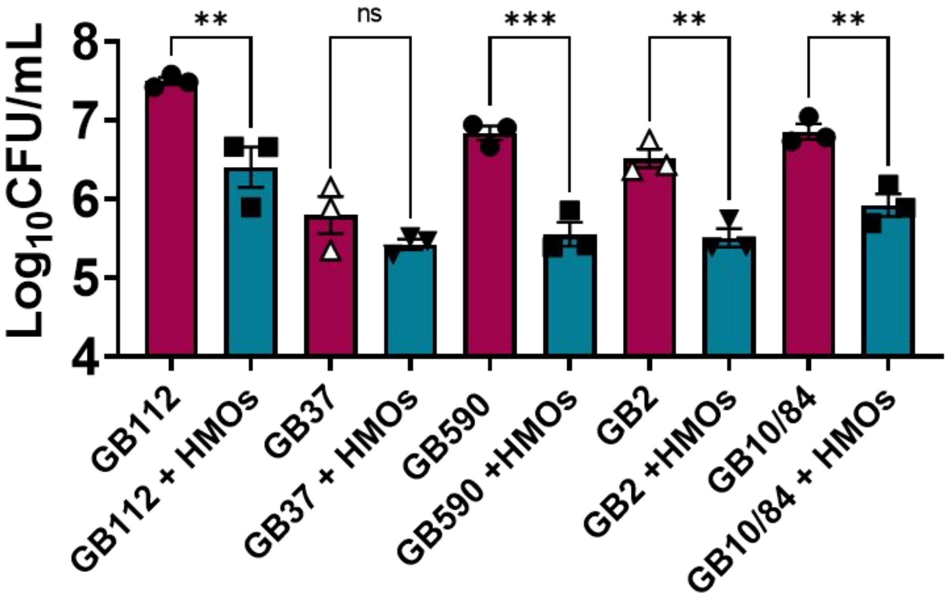

Figure S3. Human milk oligosaccharides reduce bacterial adherence to gestational membranes. Bacterial adherence was evaluated by quantitative culture (Log CFU/mL) in response to HMO supplementation. Data displayed represent ± SEM of three independent experiments, each with two technical replicates. In the presence of 5 mg/mL of HMOs, four strains of GBS exhibited significantly diminished adherence as determined by Student's t-test **P < 0.01, ***P < 0.001, and ****P < 0.0001).

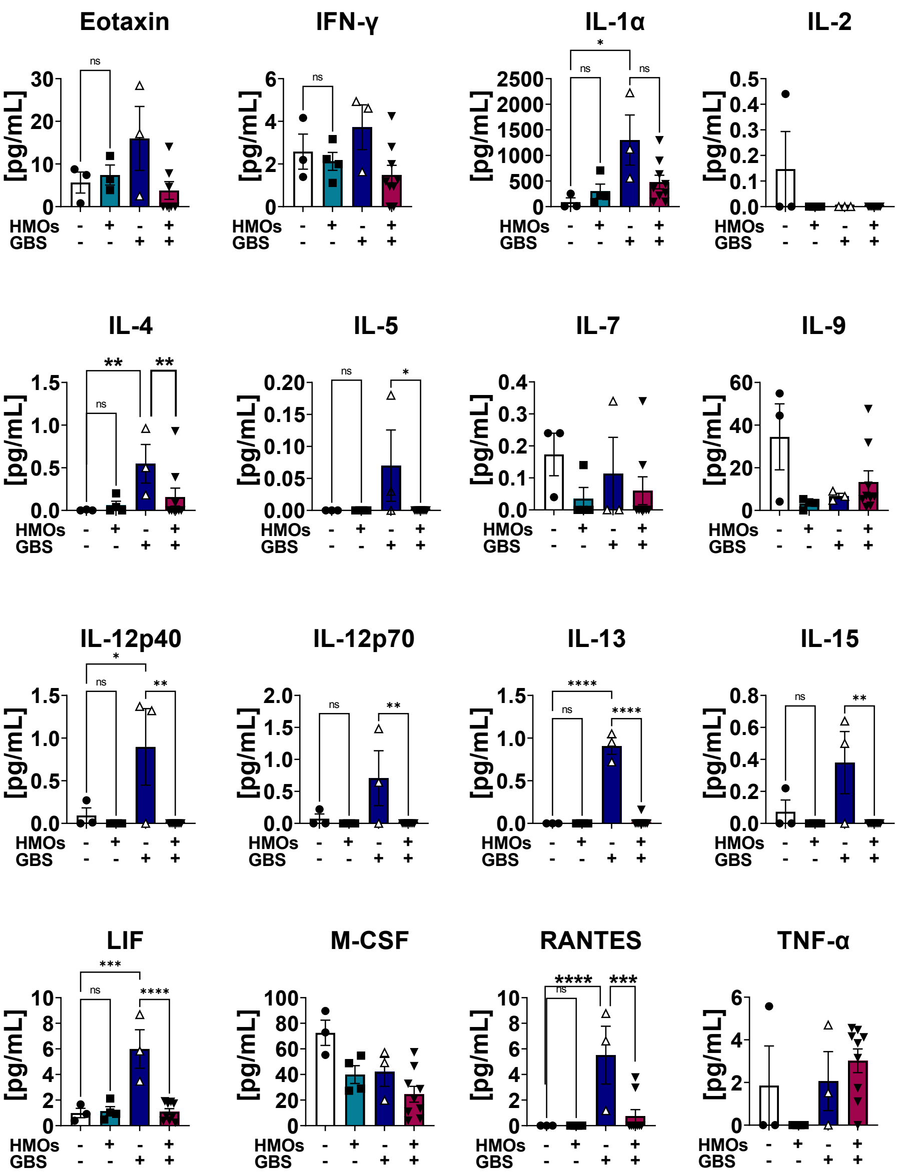

Figure S4. Analysis of cytokine production in decidual tissue in response to GBS infection. Multiplex cytokine analyses of decidua tissues after ascending vaginal infection with wild-type GB590 (dark blue bars), wild-type GB590 with 5 mg/mL HMOs (magenta), or the uninfected controls either with 5 mg/mL HMOs (teal bars) or untreated (white bars). Decidua tissues were collected from pregnant mice on embryonic day E15.5, two days post- vaginal infection with GBS. Graphs indicate quantification of eotaxin, IFN-γ, IL-1α, IL-2, IL-3, IL-4, IL-5, IL-7, IL-9, IL-10, IL-12p40, IL-12p70, IL-13, IL-15, IL-17, LIF, M-CSF, RANTES, TNF-α, and VEGF levels. Bars indicate mean values +/- standard error mean with individual data points representing results from decidual tissues from individual dams. *P<0.05, **P<0.01, ***P<0.001, ****P<0.0001, by Outliers test with either one-way ANOVA with Tukey's post-hoc multiple comparisons test or by two-tailed Student's *t* test with Welch's corrections test. NS= not statistically significant. Results indicate that HMO supplementation reverses the full initiation of proinflammatory cytokine response in decidua tissues in an ascending model of infection during pregnancy.

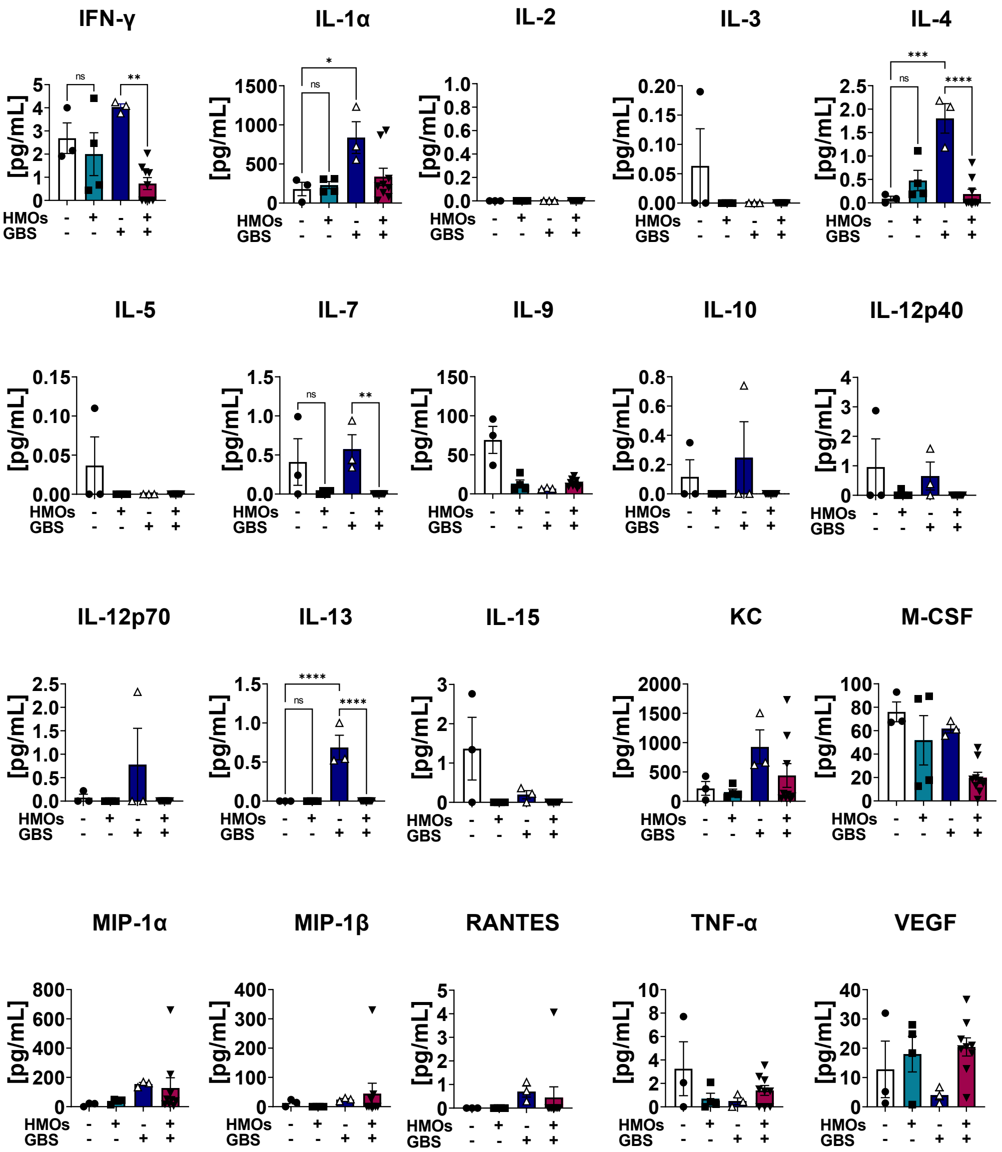

Figure S5. Analysis of cytokine production in placenta tissue in response to GBS infection. Multiplex cytokine analyses of placenta tissues after ascending vaginal infection with wild-type GB590 (dark blue bars), wild-type GB590 with 5 mg/mL HMOs (magenta), or the uninfected controls either with 5 mg/mL HMOs (teal bars) or untreated (white bars). Placenta tissues were collected from pregnant mice on embryonic day E15.5, two days post- vaginal infection with GBS. Graphs indicate quantification of eotaxin, IFN-γ, IL-1α, IL-2, IL-3, IL-4, IL-5, IL-7, IL-9, IL-10, IL-12p40, IL-12p70, IL-13, IL-15, IL-17, LIF, M-CSF, RANTES, TNF-α, and VEGF levels. Bars indicate mean values +/- standard error mean with individual data points representing results from placental tissues from individual dams. *P<0.05, **P<0.01, ***P<0.001, ****P<0.0001, by one-way ANOVA with Tukey's post-hoc multiple comparisons test. NS= not statistically significant. Results indicate that HMO supplementation reverses the full initiation of proinflammatory cytokine response in placental tissues in an ascending model of infection during pregnancy.

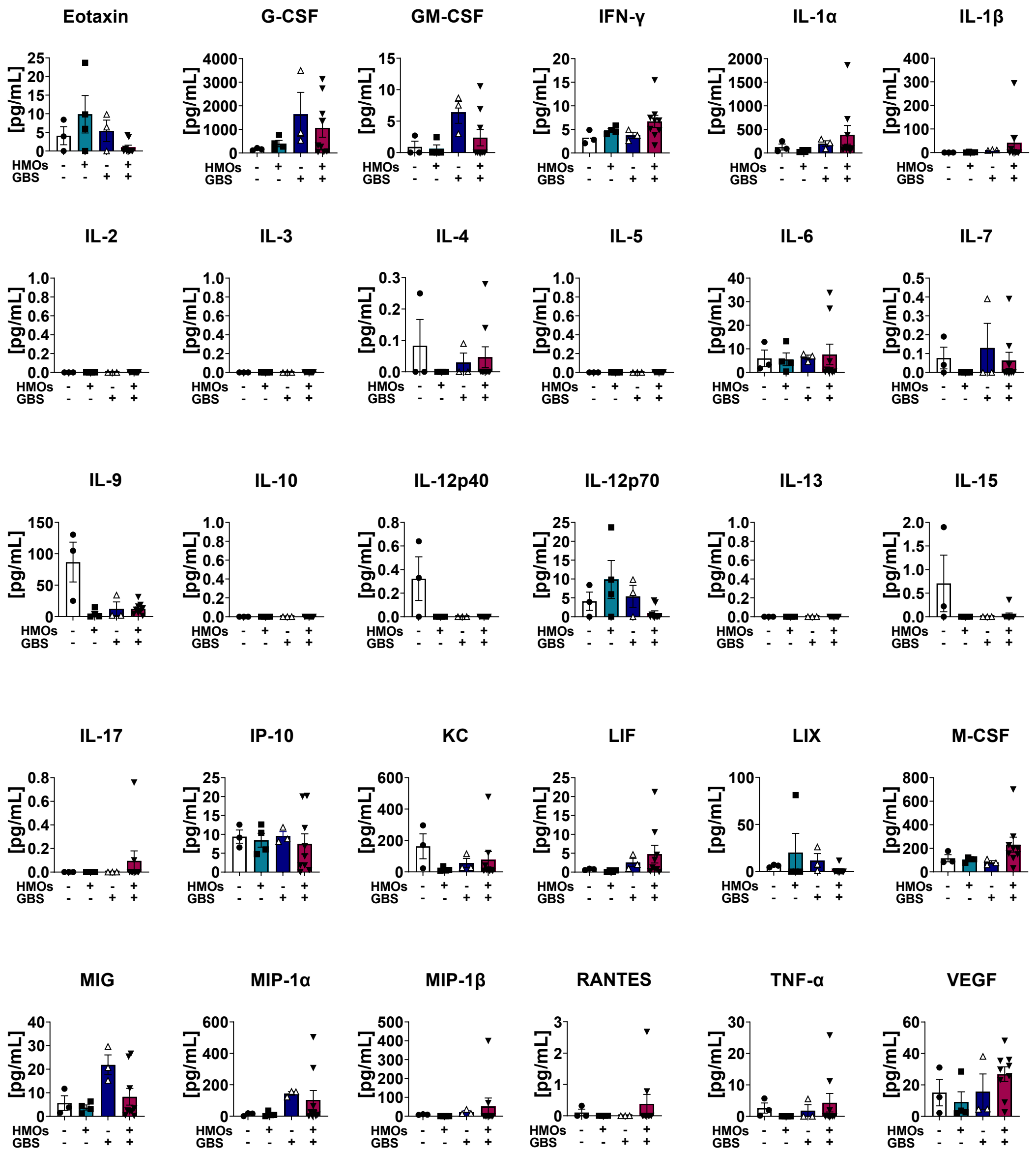

Figure S6. Analysis of cytokine production in amnion tissue in response to GBS infection. Multiplex cytokine analyses of fetal tissues after ascending vaginal infection with wild-type GB590 (dark blue bars), wild-type GB590 with 5 mg/mL HMOs (magenta), or the uninfected controls either with 5 mg/mL HMOs (teal bars) or untreated (white bars). Amnion tissues were collected from pregnant mice on embryonic day E15.5, two days post- vaginal infection with GBS. Graphs indicate quantification of eotaxin, G-CSF, GM-CSF, IFN-γ, IL-1α, IL-1β, IL-2, IL-3, IL-4, IL-5,IL-6, IL-7, IL-9, IL-10, IL-12p40, IL-12p70, IL-13, IL-15, IL-17, IP-10, KC, LIF, LIX, M-CSF, MIG, MIP-1α, MIP-1β, RANTES, TNF-α, and VEGF levels. Bars indicate mean values +/- standard error mean with individual data points representing results from amnion tissues from individual dams. *P<0.05, **P<0.01, ***P<0.001, ****P<0.0001, by one-way ANOVA with Tukey's post-hoc multiple comparisons test. NS= not statistically significant. Results indicate that HMO supplementation reverses the full initiation of proinflammatory cytokine response in amnion tissues in an ascending model of infection during pregnancy.

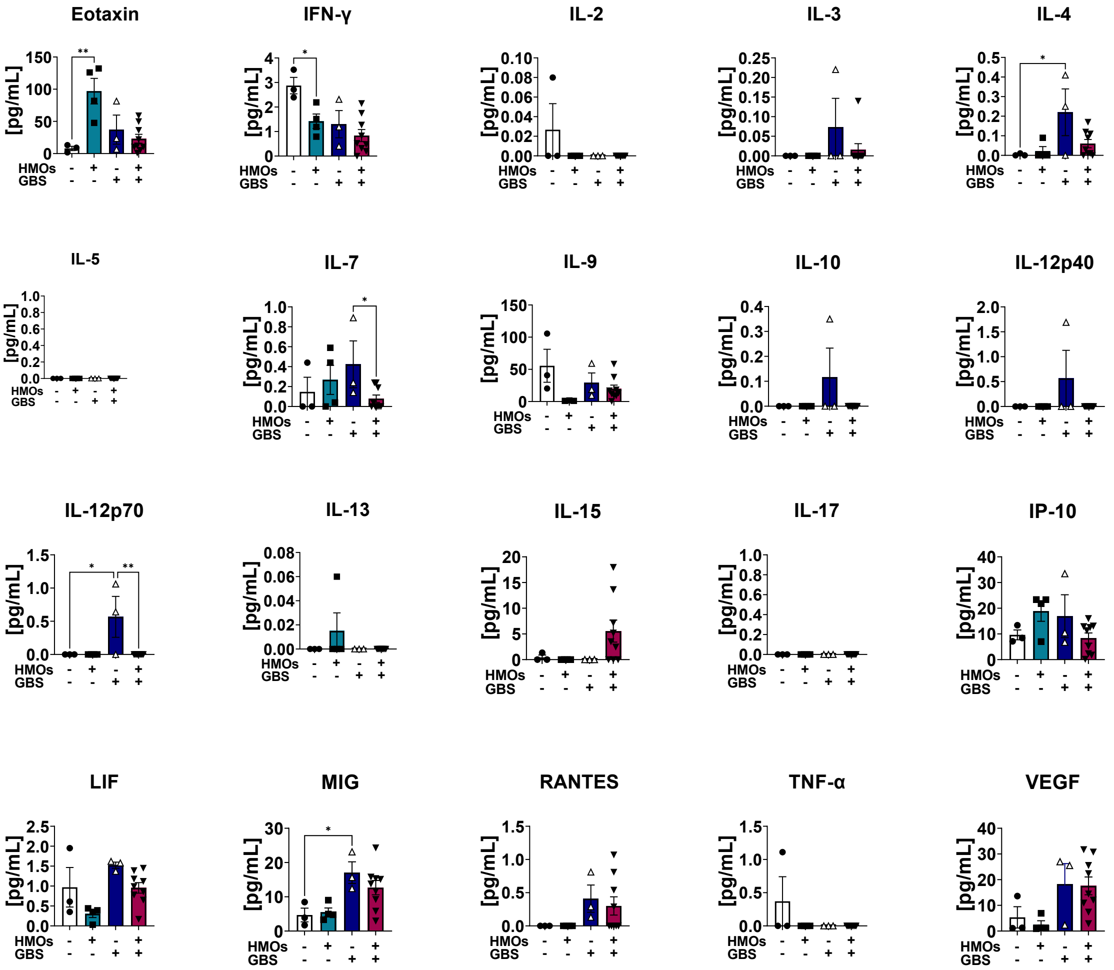

Figure S7. Analysis of cytokine production in fetal tissue in response to GBS infection. Multiplex cytokine analyses of fetal tissues after ascending vaginal infection with wild-type GB590 (dark blue bars), wild-type GB590 with 5 mg/mL HMOs (magenta), or the uninfected controls either with 5 mg/mL HMOs (teal bars) or untreated (white bars). Fetal tissues were collected from pregnant mice on embryonic day E15.5, two days post- vaginal infection with GBS. Graphs indicate quantification of eotaxin, IFN-γ, IL-2, IL-3, IL-4, IL-5, IL-7, IL-9, IL-10, IL-12p40, IL-12p70, IL-13, IL-15, IL-17, IP-10, LIF, MIG, RANTES, TNF-α, and VEGF levels. Bars indicate mean values +/- standard error mean with individual data points representing results from fetal tissues from different dams. *P<0.05, **P<0.01, ***P<0.001, ****P<0.0001, by one-way ANOVA with Tukey's post-hoc multiple comparisons test. NS= not statistically significant. Results indicate that HMO supplementation reverses the full initiation of proinflammatory cytokine response in fetal tissues in an ascending model of infection during pregnancy.

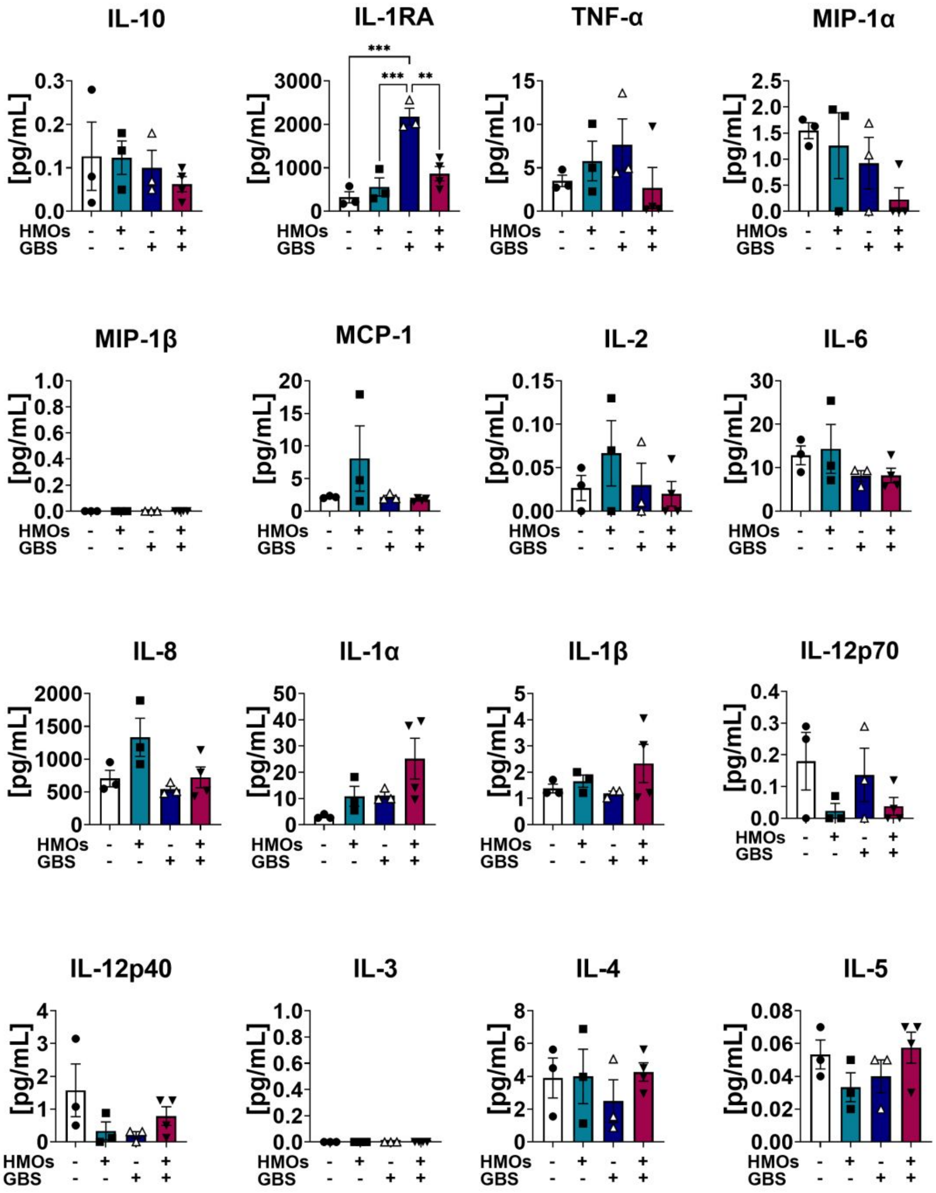

Figure S8. Analysis of cytokine production in EpiVaginal™ tissues in response to GBS infection. Multiplex cytokine analyses of organoid after ascending vaginal infection with wild-type GB590 (dark blue bars), wild-type GB590 with 5 mg/mL HMOs (magenta), or the uninfected controls either with 5 mg/mL HMOs (teal bars) or untreated (white bars). Graphs indicate quantification of IL-10, IL-IRα, TNF-α, MIP-1α, MIP-1β, MCP-1, IL-2, IL-6, IL-8, IL-1α, IL-1β, IL-12p70, IL-12p40, IL-3, IL-4, and IL-5 levels. Bars indicate mean values +/- standard error mean with individual data points representing results from separate biological replicates. **P<0.01 and ***P<0.001, by one-way ANOVA with Tukey's post-hoc multiple comparisons test. NS= not statistically significant.

Table S3. Comparison of cytokines displaying a phenotype in response to HMO treatment.

	Tissue Compartment			
	**Decidua**	**Fetus**	**Placenta**	**Amnion**
**Cytokines**	MCP-1	MCP-1	MCP-1	MCP-1
	MIP-2	MIP-2	MIP-2	MIP-2
	IL-1β	IL-1β	IL-1β	
	IL-6	IL-6	IL-6	
	LIX	LIX	LIX	
	KC	KC		
	MIP-1α	MIP-1α		
	IP-10		IP-10	
	MIG		MIG	
	GM-CSF	GM-CSF	GM-CSF	
	MIP-1β	MIP-1β		
	G-CSF		G-CSF	
		IL-1α		
		M-CSF		
			IL-17	
			LIF	
			Eotaxin	

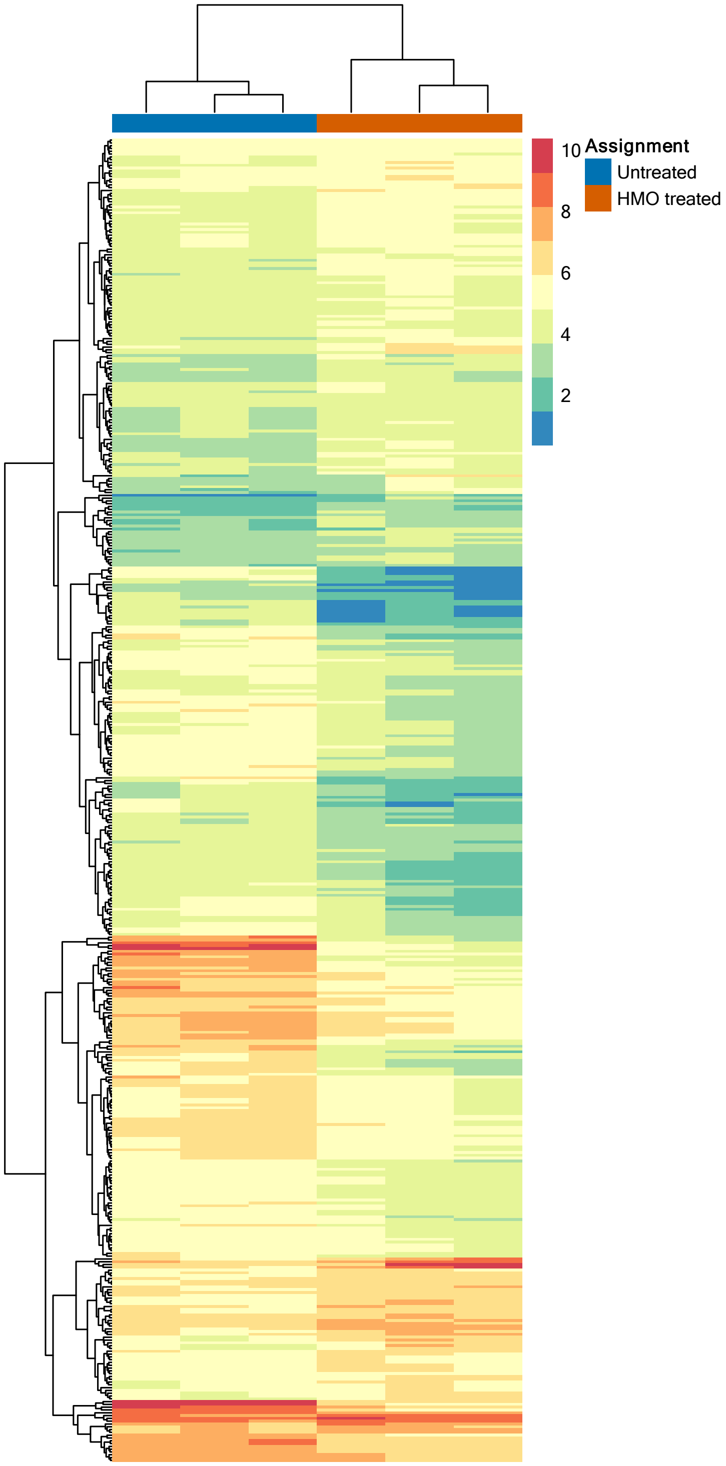

Figure S9. Heatmap of 474 significant transcripts identified with DESeq2. The level of expression is shown as the logarithm of TPMs. Medium alone control (untreated, blue) versus HMO- treated samples (HMO, red) are shown. Columns indicate three independent biological replicates utilized for analysis.

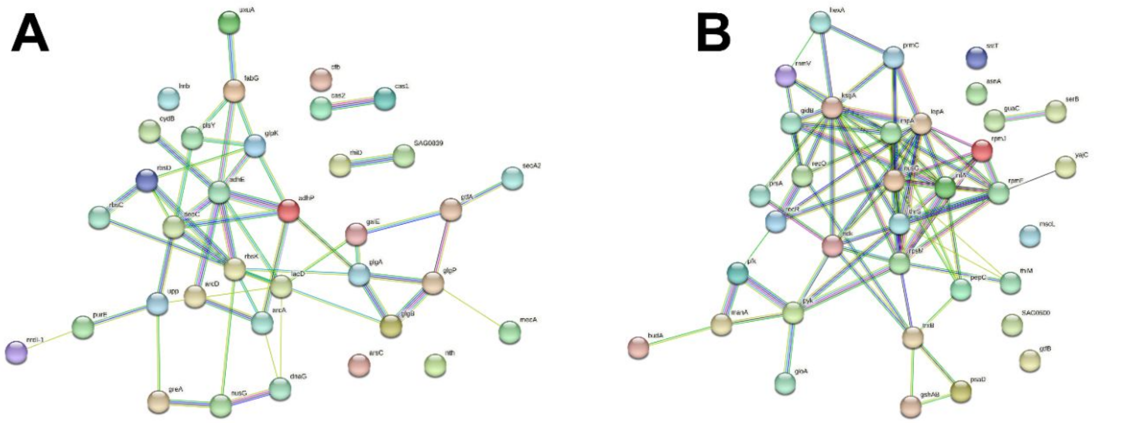

Figure S10. STRING analysis of potential protein-protein interactions in GBS in the presence or absence of HMOs. Transcripts that were significantly more abundant in cells grown in medium alone versus cells grown in the presence of HMOs were analyzed by STRING software (https://string-db.org/) which revealed that GBS gene expression in medium alone is highly enriched for networks associated with carbohydrate (specifically glucose) metabolism (A). Conversely, in the presence of HMOs, differential gene expression shifts towards networks enriched for nitrogen metabolism (B).

Table S4. STRING analyses reveals predicted protein-protein-interactions implicating functional enrichment of specific metabolic pathways.

**Culture Conditions**	**Functional Enrichment for Pathway**	**P-values calculated using Benjamini-Hochberg procedure with multiple corrections test**
Medium Alone	Carbohydrate catabolism	0.0326
Medium Alone	Carbohydrate metabolism	0.0059
Medium Alone	D-ribose and pentose catabolism	0.0220
Medium Alone	Glycogen metabolism	0.0220
Medium Alone	Mixed carbohydrate metabolism	0.0160
Medium Alone	Carbohydrate and pyruvate metabolism	0.0199
Medium +HMOs	Gene expression	0.0364
Medium +HMOs	Organonitrogen compound biosynthesis	0.0440
Medium +HMOs	Organonitrogen metabolism	0.0034
Medium +HMOs	Cellular nitrogen metabolism	0.0014
Medium +HMOs	Nitrogen compound metabolism	0.00013
Medium +HMOs	Translation, and RNA metabolism	0.0472

References

1. Ackerman, D. L.; Doster, R. S.; Weitkamp, J. H.; Aronoff, D. M.; Gaddy, J. A.; Townsend, S. D., Human Milk Oligosaccharides Exhibit Antimicrobial and Antibiofilm Properties against Group B *Streptococcus*. *ACS Infect Dis* **2017,** *3* (8), 595-605.
2. Chambers, S. A.; Gaddy, J. A.; Townsend, S. D., Synthetic Ellagic Acid Glycosides Inhibit Early Stage Adhesion of *Streptococcus agalactiae* Biofilms as Observed by Scanning Electron Microscopy. *Chem Eur J* **2020,** *26* (44), 9923-9928.
3. Gaddy, J. A.; Radin, J. N.; Cullen, T. W.; Chazin, W. J.; Skaar, E. P.; Trent, M. S.; Algood, H. M., *Helicobacter pylori* Resists the Antimicrobial Activity of Calprotectin via Lipid A Modification and Associated Biofilm Formation. *mBio* **2015,** *6* (6), e01349-15.
4. Gaddy, J. A.; Tomaras, A. P.; Actis, L. A., The *Acinetobacter baumannii* 19606 OmpA protein plays a role in biofilm formation on abiotic surfaces and in the interaction of this pathogen with eukaryotic cells. *Infect Immun* **2009,** *77* (8), 3150-60.
5. Kothary, V.; Doster, R. S.; Rogers, L. M.; Kirk, L. A.; Boyd, K. L.; Romano-Keeler, J.; Haley, K. P.; Manning, S. D.; Aronoff, D. M.; Gaddy, J. A., Group B *Streptococcus* Induces Neutrophil Recruitment to Gestational Tissues and Elaboration of Extracellular Traps and Nutritional Immunity. *Front Cell Infect Microbiol* **2017,** *7*, 19.
6. Randis, T. M.; Gelber, S. E.; Hooven, T. A.; Abellar, R. G.; Akabas, L. H.; Lewis, E. L.; Walker, L. B.; Byland, L. M.; Nizet, V.; Ratner, A. J., Group B *Streptococcus* β-hemolysin/cytolysin breaches maternal-fetal barriers to cause preterm birth and intrauterine fetal demise *in vivo*. *J Infect Dis* **2014,** *210* (2), 265-73.
7. Doster, R. S.; Kirk, L. A.; Tetz, L. M.; Rogers, L. M.; Aronoff, D. M.; Gaddy, J. A., *Staphylococcus aureus* Infection of Human Gestational Membranes Induces Bacterial Biofilm Formation and Host Production of Cytokines. *J Infect Dis* **2017,** *215* (4), 653-657.
8. Korir, M. L.; Doster, R. S.; Lu, J.; Guevara, M. A.; Spicer, S. K.; Moore, R. E.; Francis, J. D.; Rogers, L. M.; Haley, K. P.; Blackman, A., et al., *Streptococcus agalactiae* cadD alleviates metal stress and promotes intracellular survival in macrophages and ascending infection during pregnancy. *Nat Commun* **2022,** *13* (1), 5392.
9. Kopylova, E.; Noé, L.; Touzet, H., SortMeRNA: fast and accurate filtering of ribosomal RNAs in metatranscriptomic data. *Bioinformatics* **2012,** *28* (24), 3211-3217.
10. Dobin, A.; Davis, C. A.; Schlesinger, F.; Drenkow, J.; Zaleski, C.; Jha, S.; Batut, P.; Chaisson, M.; Gingeras, T. R., STAR: ultrafast universal RNA-seq aligner. *Bioinformatics* **2013,** *29* (1), 15-21.
11. Szklarczyk, D.; Gable, A. L.; Lyon, D.; Junge, A.; Wyder, S.; Huerta-Cepas, J.; Simonovic, M.; Doncheva, N. T.; Morris, J. H.; Bork, P., et al., STRING v11: protein-protein association networks with increased coverage, supporting functional discovery in genome-wide experimental datasets. *Nucleic Acids Res* **2019,** *47* (D1), D607-d613.
